# Supplementary material for: Synthesis and Characterization of Metallopolymer Networks Featuring Triple Shape-Memory Ability Based on Different Reversible Metal Complexes
Source: Polymers (Basel). 2022 Apr 29;14(9):1833. doi: 10.3390/polym14091833 (PMC9105372; doi:10.3390/polym14091833)
Supplement: Supplementary file 1 [file polymers-14-01833-s001.zip › polymers-1686489-supplementary.pdf]

polymers

MDPI

Supplementary materials: Synthesis and characterization of metallopolymer networks featuring triple shape-memory ability based on different reversible metal complexes

Josefine Meurer [1,2], Thomas Bätz [1,2], Julian Hniopek [3,4,5], Milena Jäger [1,2], Stefan Zechel [1,2], Michael Schmitt [3,4], Jürgen Popp [3,4,5], Martin D. Hager [1,2] and Ulrich S. Schubert [1,2,4*]

1 Laboratory of Organic and Macromolecular Chemistry (IOMC), Friedrich Schiller University Jena, Humboldtstr. 10, 07743 Jena, Germany; josefine.meurer@uni-jena.de (J.M.); thomas.baetz@uni-jena.de (T.B.); milena.jaeger@uni-jena.de (M.J.); stefan.zechel@uni-jena.de (S.Z.); martin.hager@uni-jena.de (M.D.H.)
2 Jena Center of Soft Matter (JCSM), Friedrich Schiller University Jena, Philosophenweg 7, 07743 Jena, Germany
3 Institute of Physical Chemistry (IPC), Friedrich Schiller University Jena, Helmholtzweg 4, 07743 Jena, Germany; julian.hniopek@uni-jena.de (J.H.); m.schmitt@uni-jena.de (M.S.); juergen.popp@uni-jena.de (J.P.)
4 Abbe Center of Photonics, Friedrich Schiller University Jena, Albert-Einstein-Straße 6, 07745 Jena, Germany
5 Leibniz Institute of Photonic Technology, e. V. Jena, Albert-Einstein-Str. 9, 07745 Jena, Germany
* Correspondence: ulrich.schubert@uni-jena.de

Contents

Synthesis of the monomers 2

Synthesis of 6-(2,2':6'2''-terpyridin-4'-yloxy)-hexyl methacrylate (**Tpy-MA**) 2

Synthesis of 1-[4-(pyridine-2-yl)-1*H*-1,2,3-triazol-1-yl]undecanyl-acetate (**Triaz-Py**) and 11-[4-(pyridine-2-yl)-1*H*-1,2,3-triazol-1-yl]undecanyl-methacrylate (**Tiaz-Py-MA**) 2

Isothermal titration calorimetry (ITC) 2

Synthesis of model polymers 4

Synthesis of model metallopolymer networks 7

Nuclear magnetic resonance spectroscopy (NMR) 8

Differential scanning calorimetry (DSC) 11

Thermogravimetric analysis (TGA) 12

Thermo-mechanical analyses (TMA) 13

Investigation of the dual shape-memory properties 14

Investigation of the triple shape-memory properties 18

Photo series of the triple shape-memory test .... **Error! Bookmark not defined.**

FT-Raman spectroscopy 22

References 24

Synthesis of the monomers

Synthesis of 6-(2,2′:6′2′′-terpyridin-4′-yloxy)-hexyl methacrylate (Tpy-MA)

The synthesis of 6-(2,2′:6′2′′-terpyridin-4′-yloxy)-hexyl methacrylate (**Tpy-MA**) was performed according to a literature procedure [1]. The reaction scheme is displayed in **Scheme S1**.

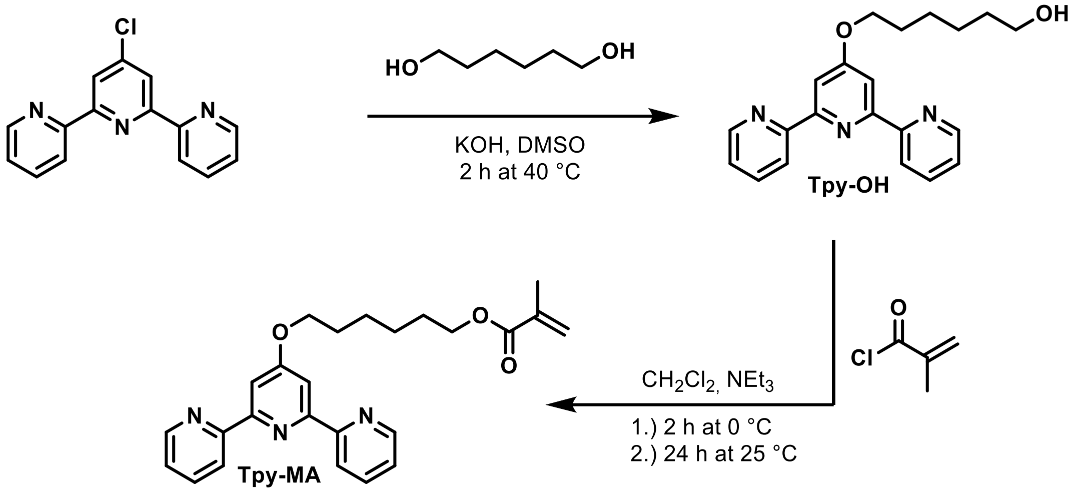

Scheme 1. Reaction scheme for the synthesis of the terpyridine monomer 6-(2,2′:6′2′′-terpyridin-4′-yloxy)-hexyl methacrylate (**Tpy-MA**) according to literature [1].

Synthesis of 1-[4-(pyridine-2-yl)-1H-1,2,3-triazol-1-yl]undecanyl-acetate (Triaz-Py) and 11-[4-(pyridine-2-yl)-1H-1,2,3-triazol-1-yl]undecanyl-methacrylate (Tiaz-Py-MA)

The syntheses of 1-[4-(pyridine-2-yl)-1*H*-1,2,3-triazol-1-yl]undecanyl-acetate (**Triaz-Py**) and 11-[4-(pyridine-2-yl)-1*H*-1,2,3-triazol-1-yl]undecanyl-methacrylate (**Tiaz-Py-MA**) were performed according to literature procedures [2–4]. The reaction scheme is displayed in **Scheme S2**.

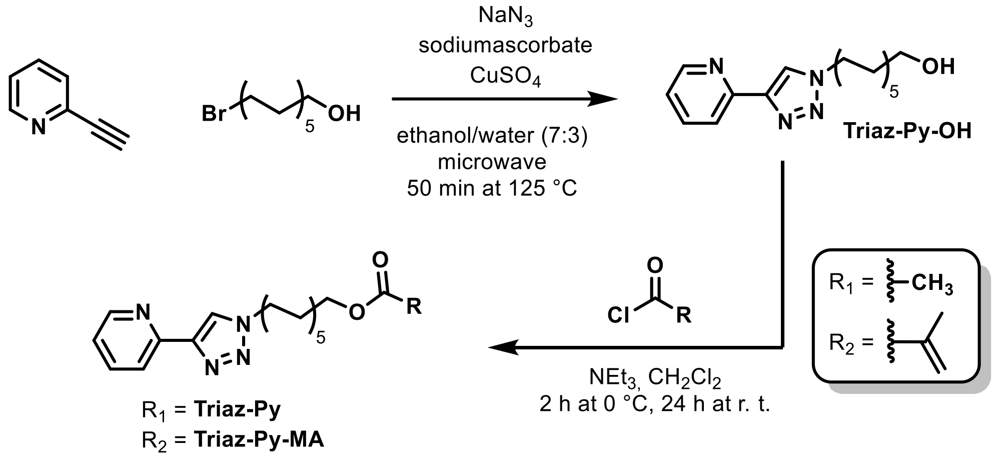

Scheme 2. Reaction scheme for the syntheses of the triazole pyridine model system 1-[4-(pyridine-2-yl)-1*H*-1,2,3-triazol-1-yl]undecanyl-acetate (**Triaz-Py**) and 11-[4-(pyridine-2-yl)-1*H*-1,2,3-triazol-1-yl]undecanyl-methacrylate (**Tiaz-Py-MA**) according to literature [2–4].

Isothermal titration calorimetry (ITC)

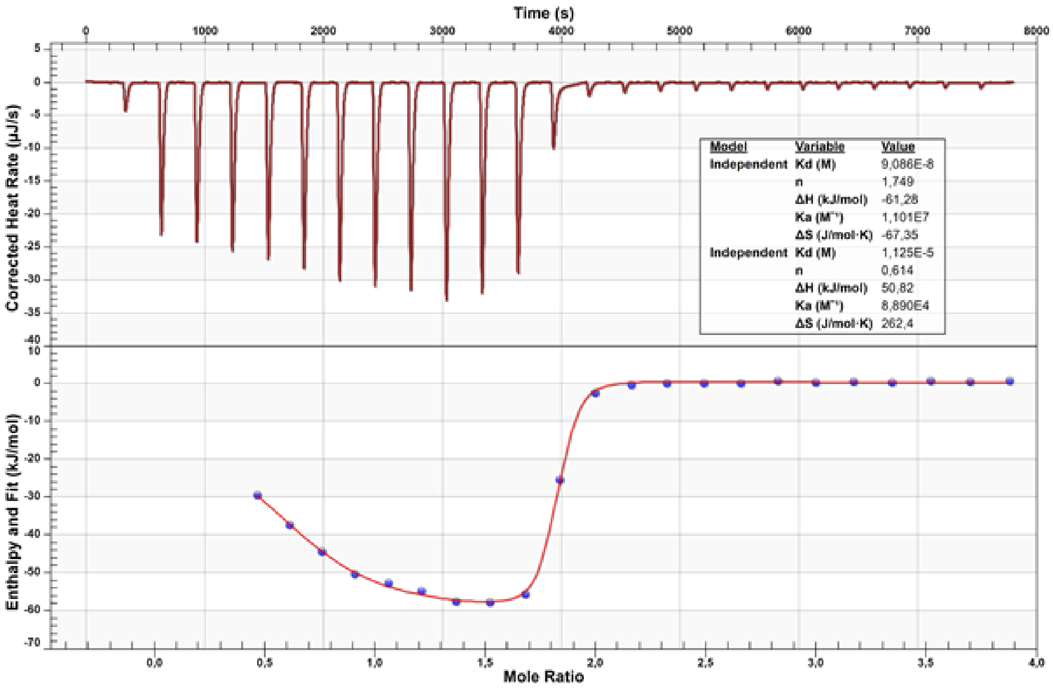

Figure S1. ITC titration data of Co(OAc)$_2$ × 4 H_2O (0.125 mM, in cell) with **Tpy** (1.70 mM, in syringe) in MeOH/$CHCl_3$ (2:1) at 303 K.

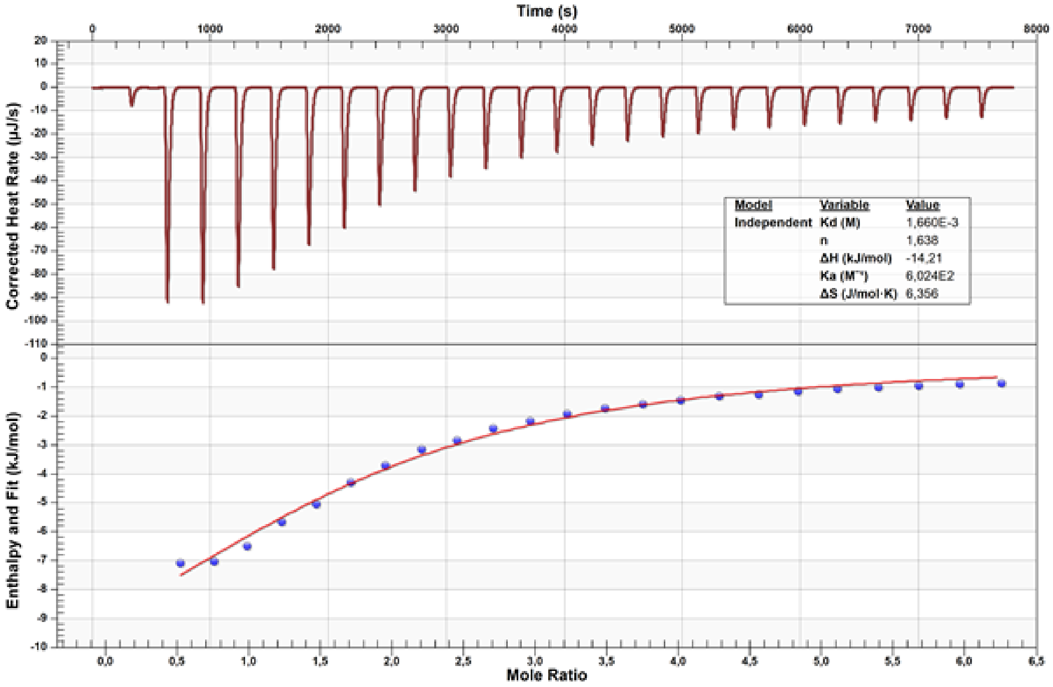

Figure S2. ITC titration data of $FeSO_4$ × 7 H_2O (1.60 mM, in cell) with **Triaz-Py** (34.01 mM, in syringe) in MeOH/$CHCl_3$ (2:1) at 303 K.

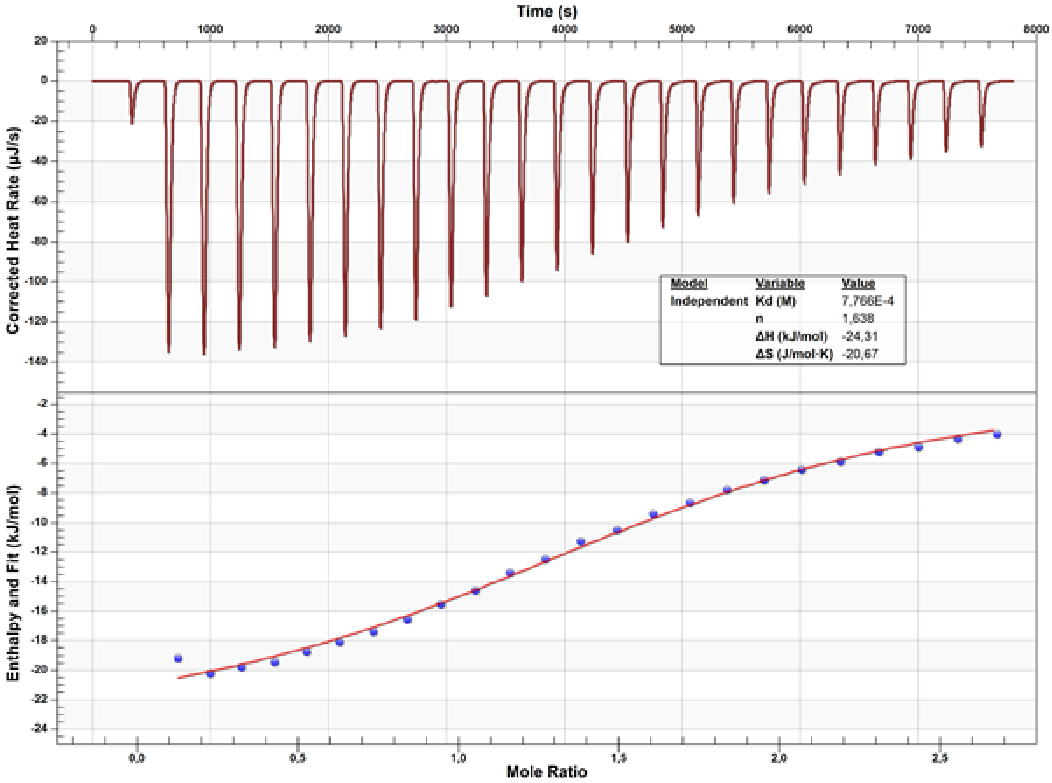

Figure S3. ITC titration data of $Co(OAc)_2 \times 4\ H_2O$ (2.81 mM in cell) with **Tiaz-Py** (25.66 mM, in syringe) in $MeOH:CHCl_3$ (2:1) at 303 K.

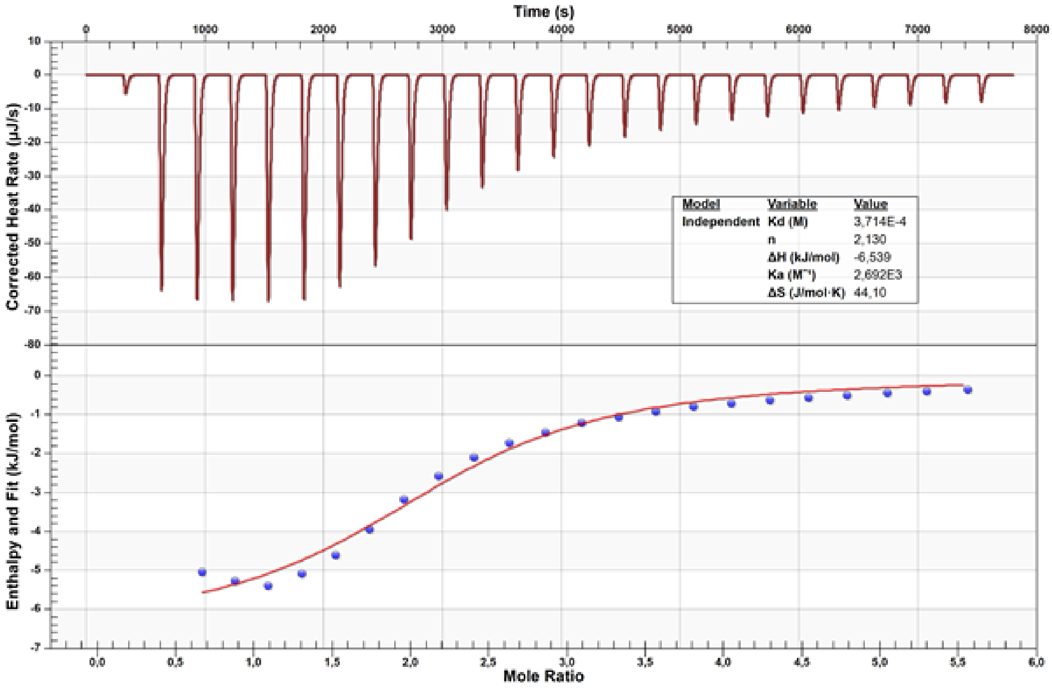

Figure S4. ITC titration data of $Zn(TFMS)_2$ (1.80 mM, in cell) with **Triaz-Py** (34.20 mM, in syringe) in $MeOH/CHCl_3$ (2:1) at 303 K.

Synthesis of model polymers

The characterization of the synthesized metallopolymer networks, which contain two different metal complexes and the covalent crosslinker at the same time, can pose some challenges within some characterization methods (e.g., FT-Raman spectroscopy). For simplification, model-metallopolymers were synthesized which only contain the main mon-

omer BMA and one of the metal complexes. Those model metallopolymers are much easier to characterize and the data obtained can be used as a reference for a better understanding of the more complex results from the networks containing two ligands and a covalent crosslinker. In the beginning, two polymers (**P2** and **P3**) each containing only one of the utilized ligand monomers were synthesized. The RAFT polymerizations were performed according to a standard procedure established in our group according to the literature and analogously to former studies [5,6]. Both polymerizations were carried out in a 250 mL one-neck-round bottom flask. As initiator 2,2′-azo*bis*(2-methylpropionitrile) (AIBN), 2-cyano-2-propyl benzodithioate (CPDB) as chain transfer agent (CTA) and furthermore one of the ligand monomers 6-(2,2′:6′2′′-terpyridin-4′-yloxy)-hexyl methacrylate (**Tpy-MA**) (in case of **P2**) or 11-[4-(pyridine-2-yl)-1*H*-1,2,3-triazol-1-yl]undecanyl-methacrylate (**Tiaz-Py-MA**) (in case of **P3**) as well as the main monomer butyl methacrylate (BMA), were placed into the flask. Subsequently, the required amount toluene was added to reach the desired concentration of 2 mol L^{-1}. The monomer to CPDB ratio was 125 to 1, the ratio of CPDT to AIBN 4 to 1. The respective utilized quantities of all substances are listed in **Table S1**. The solution was purged with nitrogen for 1 h. Afterwards, the mixture was stirred for 17 h in a preheated oil bath at 70 °C. The crude product was purified by the use of dialysis (MWCO: 3500 g mol^{-1}, THF). The solvent was changed two times each day for three days. The synthesis of **P2** and **P3** is displayed in **Scheme S3a**. The obtained polymers were characterized via size exclusion chromatography (SEC), ^{1}H NMR spectroscopy as well as elemental analysis (EA). The results are summarized in **Table S2**. The SEC investigation revealed low dispersities for both polymers indicating a successful controlled radical polymerization. Furthermore, the ^{1}H NMR spectra of the polymers were utilized to calculate the exact compositions of **P2** and **P3**. For this purpose, the aromatic signals at about 7 to 9 ppm of the ligand were related to the O-CH_2 signal of the BMA units at about 4 ppm.

P2: ^{1}H NMR (300 MHz, CD_2Cl_2) δ = 8.73 – 8.52 (m, 4H), 8.02 (d, *J* = 2.9 Hz, 4H), 7.86 (t, *J* = 7.7 Hz, 4H), 7.33 (d, *J* = 6.0 Hz, 2H), 4.19 (d, *J* = 27.4 Hz, 2H), 3.92 (s, 31H), 2.05 – 0.72 (m, 230H) ppm.

Elemental analysis: calcd. C 68.27, H 9.38, N 1.61; found. C 68.12, H 9.14, N 1.96.

P3: ^{1}H NMR (300 MHz, CD_2Cl_2) δ = 8.55 (d, *J* = 4.6 Hz, 1H), 8.25 – 8.04 (m, 2H), 7.78 (td, *J* = 7.8, 1.5 Hz, 1H), 7.33 – 7.15 (m, 1H), 4.40 (t, *J* = 7.2 Hz, 2H), 3.93 (s, 22H), 2.19 – 0.39 (m, 140H) ppm.

Elemental analysis: calcd. C 67.80, H 9.62, N 2.88; found. C 67.42, H 9.62, N 3.02.

Table S1. Utilized quantities for the synthesis of the model polymers **P2** and **P3** via RAFT-polymerization.

Model polymer		BMA	AIBN	CPDB	Ligand monomer	Toluene
	m (mg)	15 000	38.1	205.5	4 404	-
**P2** (1)	n (mmol)	105.49	0.23	0.93	10.55	-
	V (mL)	-	-	-	-	58
	m (mg)	15 000	31.1	205.5	4 056	-
**P3** (2)	n (mmol)	105.49	0.23	0.93	10.55	-
	V (mL)	-	-	-	-	58

1 Utilized ligand-monomer: **Tpy-MA**.

2 Utilized ligand-monomer: **Triaz-Py-MA**.

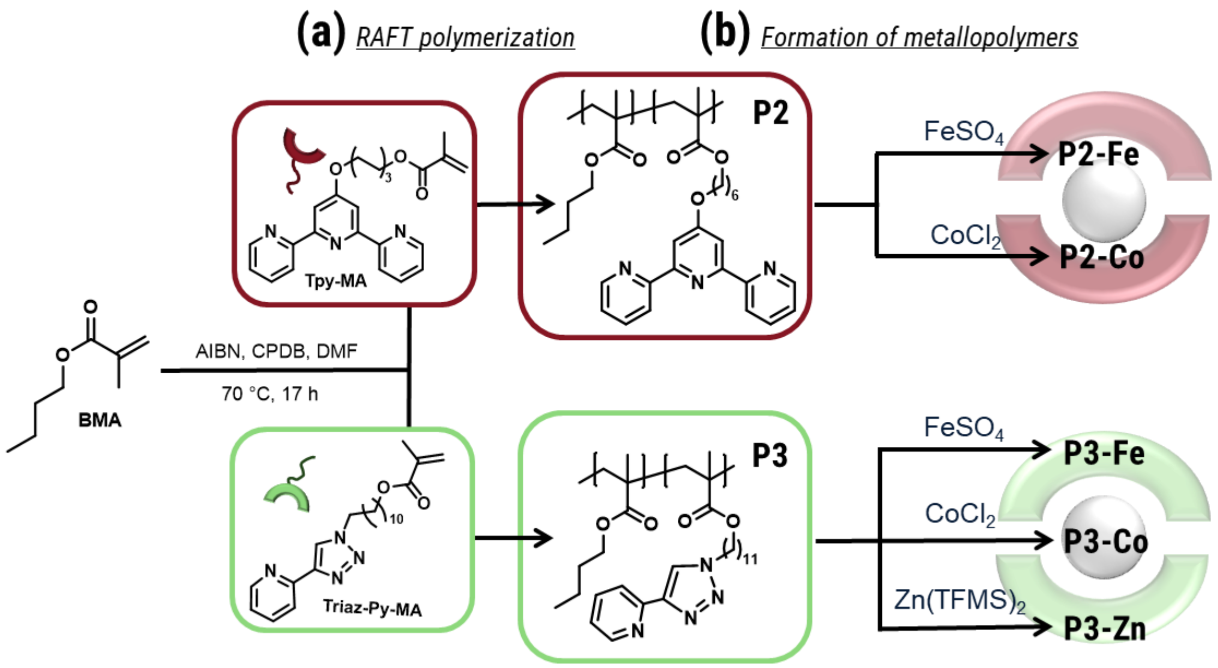

Scheme 3. Schematic representation of the synthesis of **(a)** the model polymers **P2** and **P3** via RAFT polymerization of BMA and **Tpy-MA** (**P2**) or **Triaz-Py-MA** (**P3**) and **(b)** the model metallopolymer networks **P2-Fe**, **P2-Co**, **P3-Fe**, **P3-Co,** and **P3-Zn**.

Table S2. Determined molar masses and dispersities via SEC and determined composition via ^{1}H NMR spectroscopy of the model polymers **P2** and **P3**.

**Polymer**	**SEC[1]**			**^{1}H NMR spectroscopy[2]**
	**M_n (g mol^{-1})**	**M_w (g mol^{-1})**	**Đ**	**Ligand content (%)**
**P2**	10 100	11 800	1.16	6.5
**P3**	15 200	17 100	1.13	9.1

[1] Solvent: $CHCl_3$/NEt_3/*i*-PrOH (94:4:2), PMMA standard.

[2] 300 MHz, CD_2Cl_2.

Size exclusion chromatography:

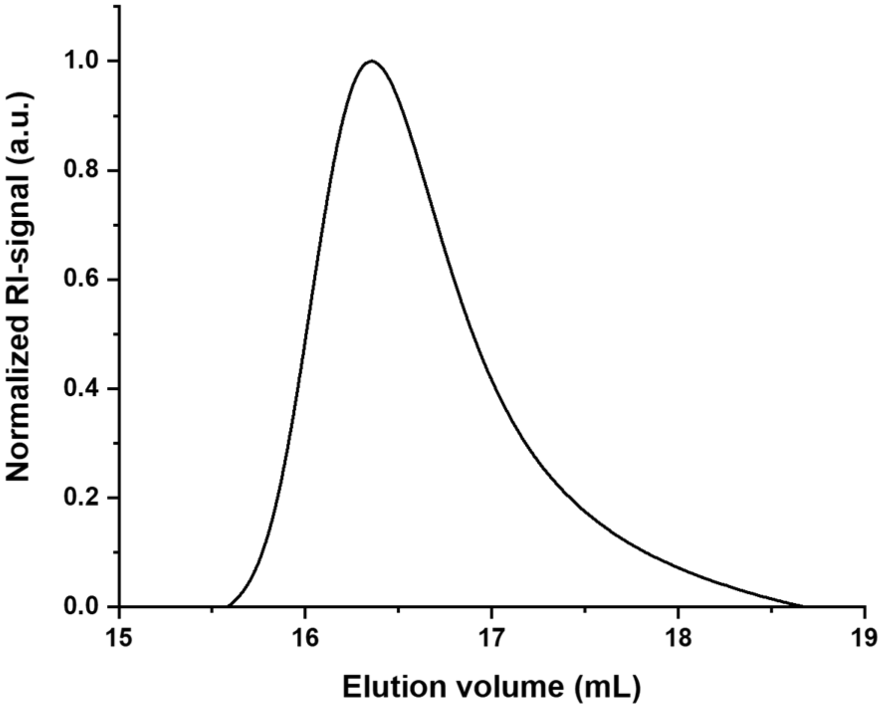

Figure S5. SEC curve of the polymer **P2** (eluent: $CHCl_3$/*i*-PrOH/NEt_3 (94:2:4), PMMA standard).

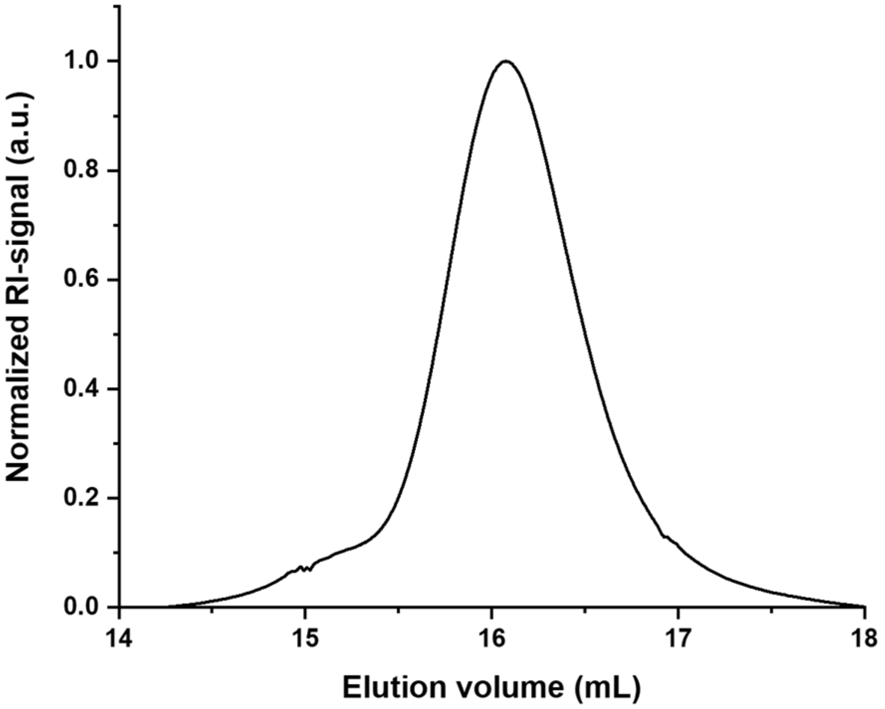

Figure S6. SEC curve of the polymer **P3** (eluent: $CHCl_3$/*i*-PrOH/NEt_3 (94:2:4), PMMA standard).

Synthesis of model metallopolymer networks

The conversion of **P2** and **P3** into metallopolymer networks was performed according to a standard procedure already presented in literature [5,6]. For the synthesis of the

model metallopolymer networks **P2-Fe**, **P2-Co**, **P3-Fe**, **P3-Co,** and **P3-Zn** the model polymer **P2**, respectively **P3,** was added to a 50 mL one-neck-round bottom flask and dissolved in chloroform (10 mL). The calculated amount of metal salt was separately dissolved in methanol (2 mL) and added to the solution of the respective polymer. The solvent was removed under reduced pressure and the resulting model metallopolymer network was dried for 24 h under vacuo at a temperature of 40 °C. The synthesis of the model metallopolymer networks is schematically displayed in **Scheme S3b**. The utilized quantities for the synthesis of all model metallopolymer networks are listed in **Table S3**.

Table S3. Utilized quantities for the synthesis of model metallopolymer networks **P2-Fe**, **P2-Co**, **P3-Fe**, **P3-Co** and **P3-Zn.**

	Polymer	m (polymer) (g)	Metal salt	m (metal salt) (mg)	n (metal salt) (mmol)
**P2-Fe**	**P2**	2.50	$FeSO_4 \times 7\ H_2O$	132	0.475
**P2-Co**		2.45	$Co(OAc)_2 \times 4\ H_2O$	117	0.470
**P3-Fe**	**P3**	2.40	$FeSO_4 \times 7\ H_2O$	171	0.615
**P3-Co**		2.48	$Co(OAc)_2 \times 4\ H_2O$	158	0.634
**P3-Zn**		2.43	$Zn(TFMS)_2$	227	0.624

Elemental analysis:

P2-Fe: calcd. C 64.88, H 9.23, N 1.53, S: 0.43; found. C 65.84, H 9.02, N 2.12, S: 0.65.
P2-Co: calcd. C 66.92, H 9.18, N 1.56; found. C 66.52, H 8.98, N 2.11.
P3-Fe: calcd. C 63.27, H 9.40, N 2.69, S: 0.56; found. C 65.15, H 9.42, N 2.91, S: 0.62.
P3-Co: calcd. C 66.03, H 9.35, N 2.75; found. C 65.64, H 9.28, N 2.89.
P3-Zn: calcd. C 62.83, H 8.84, N 2.64, S: 1.11; found. C 62.92, H 8.90, N 2.88, S: 1.21.

Nuclear magnetic resonance spectroscopy (NMR)

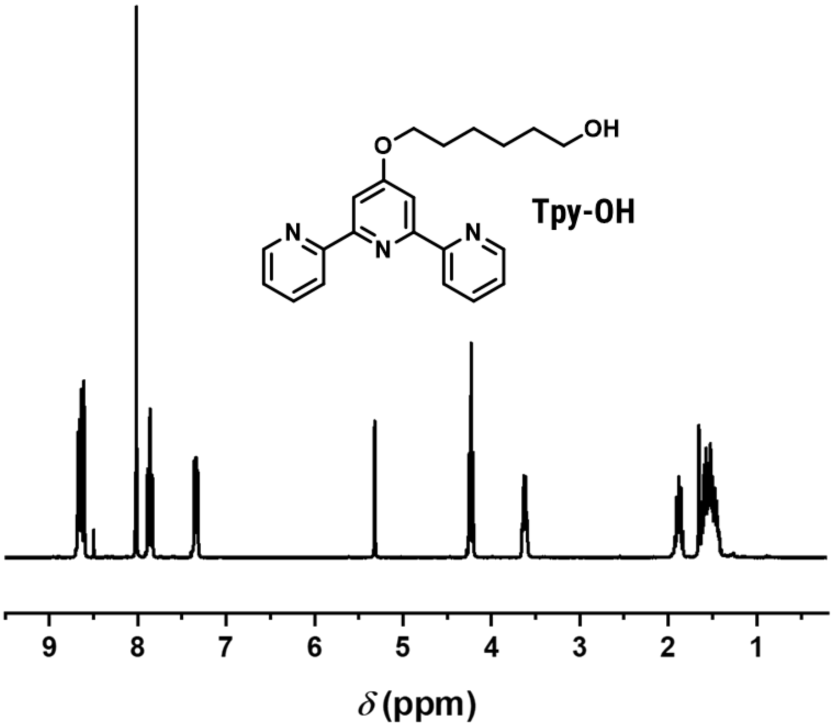

Figure S7. ^1H NMR spectrum of **Tpy-OH** (300 MHz, CD_2Cl_2).

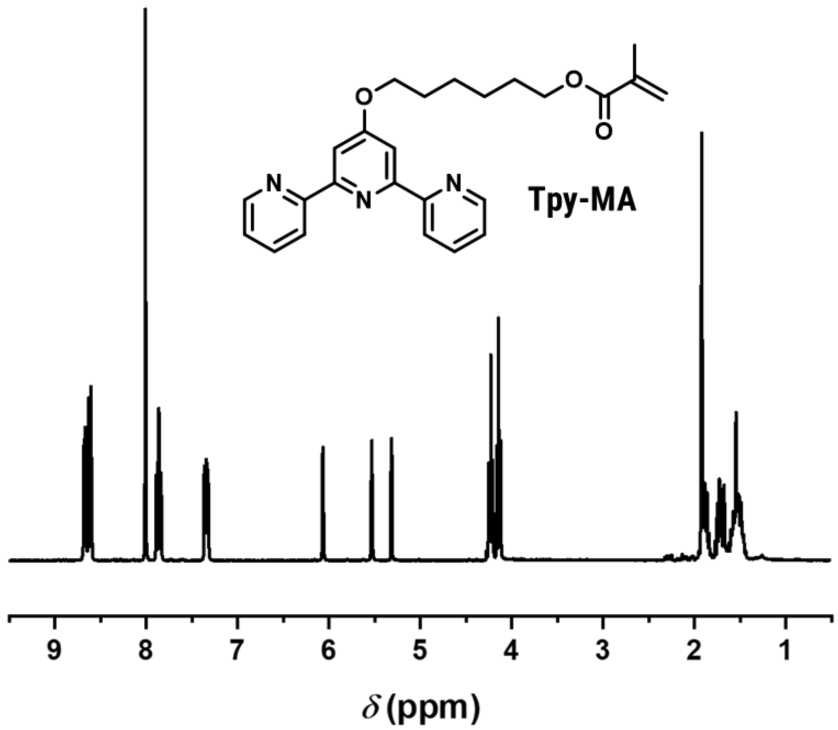

Figure S8. ^{1}H NMR spectrum of **Tpy-MA** (300 MHz, CD_2Cl_2).

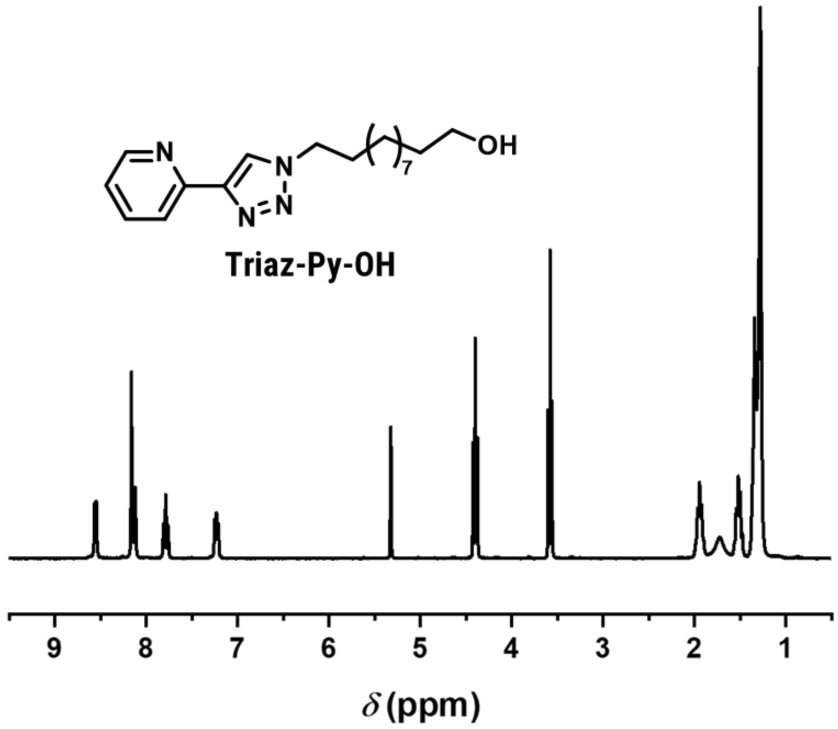

Figure S9. ^{1}H NMR spectrum of **Tiaz-Py-OH** (300 MHz, CD_2Cl_2).

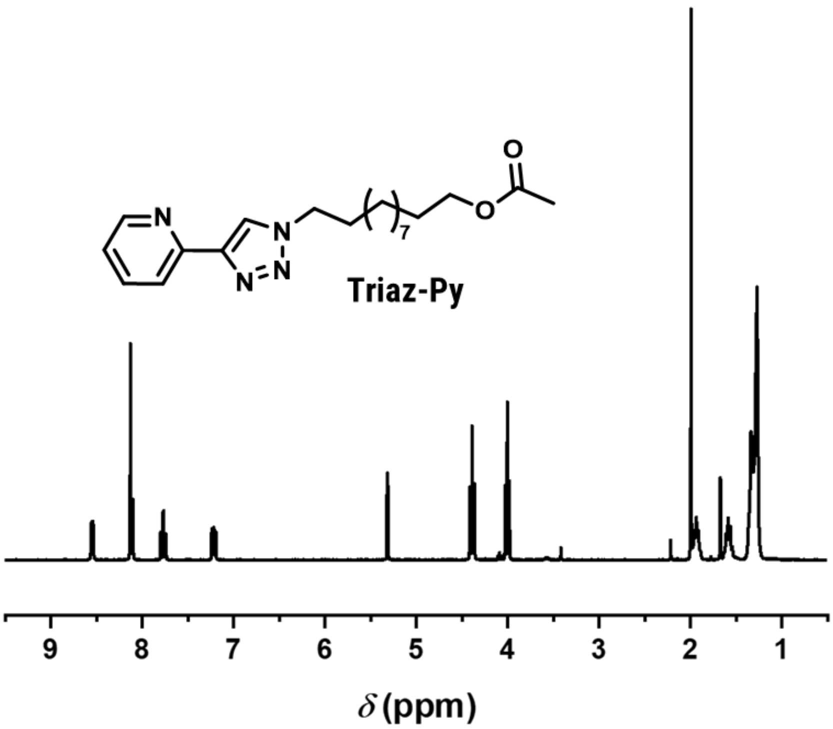

Figure S10. ^{1}H NMR spectrum of **Tiaz-Py** (300 MHz, CD_2Cl_2).

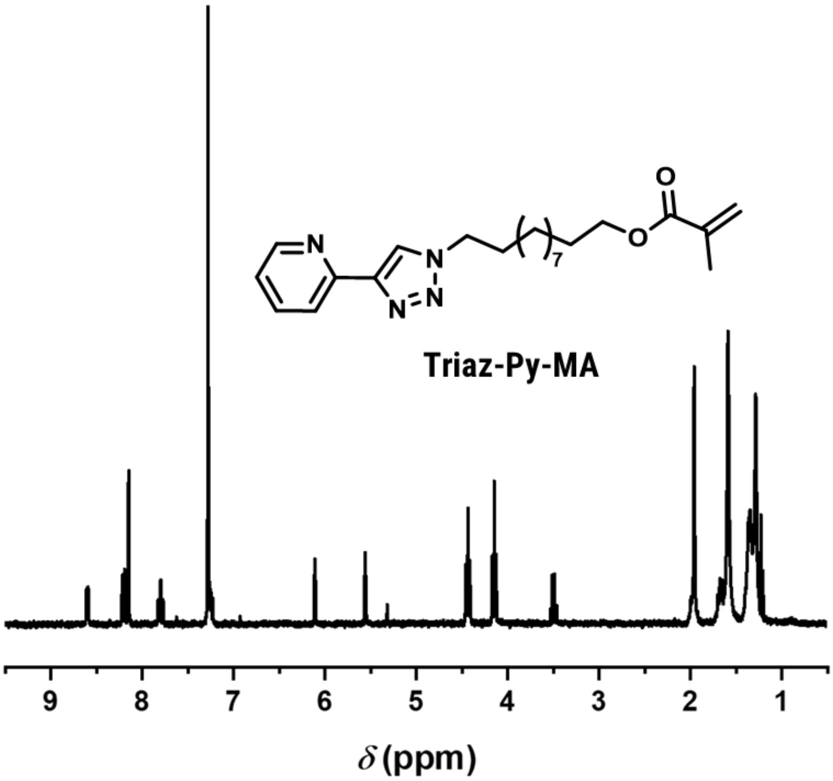

Figure S11. ^{1}H NMR spectrum of **Tiaz-Py-MA** (300 MHz, $CDCl_3$).

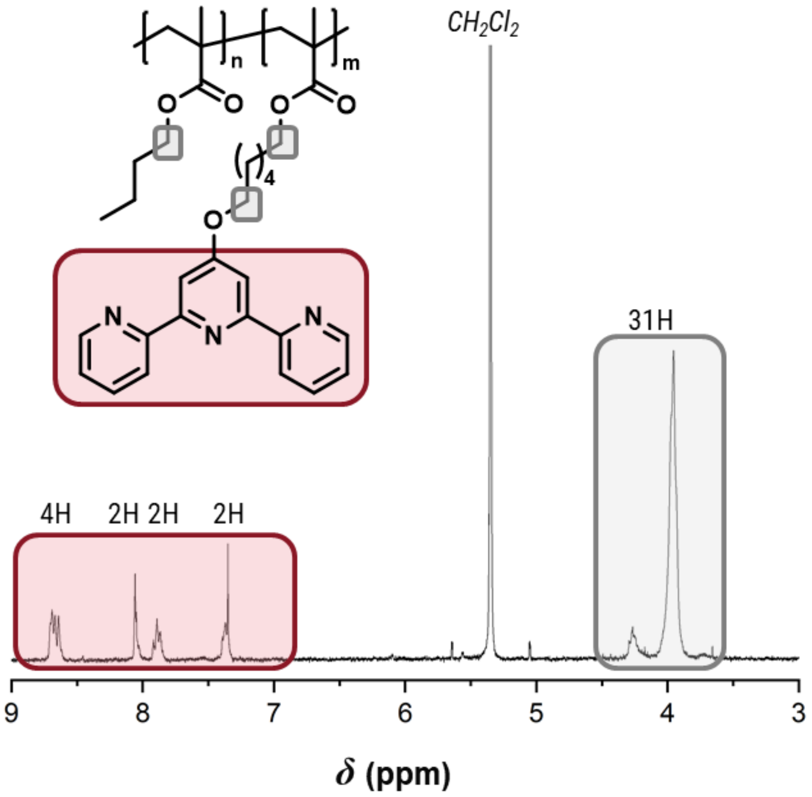

Figure S12. Zoom in to the ^{1}H NMR spectrum of **P2** (300 MHz, CD_2Cl_2).

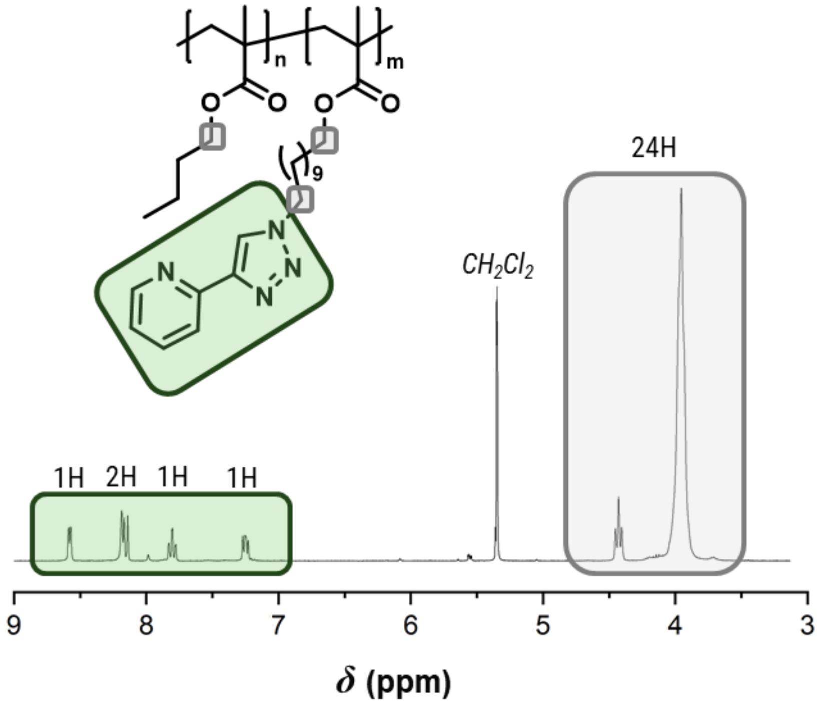

Figure S13. Zoom in to the ^{1}H NMR spectrum of **P3** (300 MHz, CD_2Cl_2).

Differential scanning calorimetry (DSC)

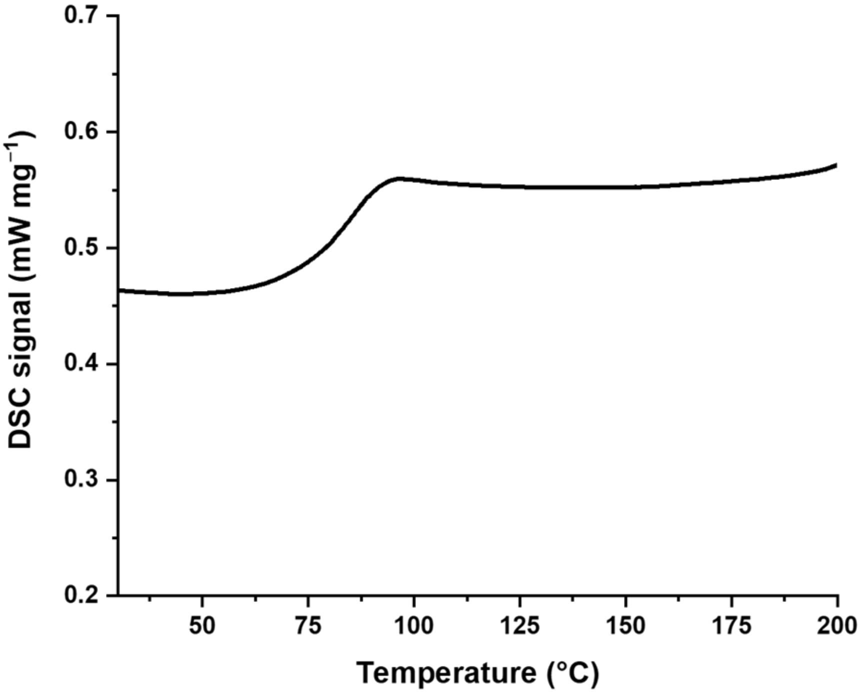

Figure S14. DSC-curve of the second heating cycle of the polymer **P1** (heating rate: 20 K min^{-1}).

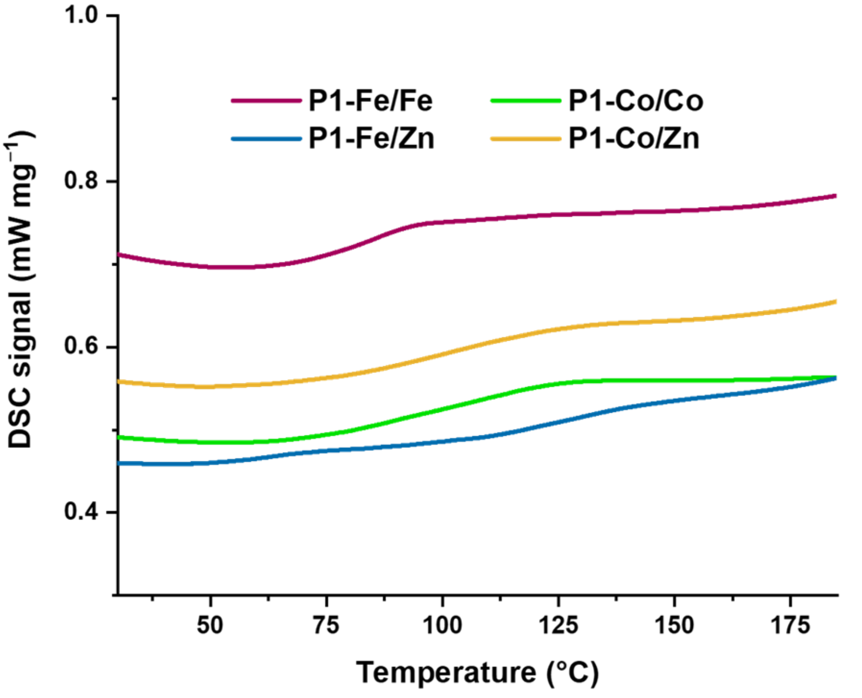

Figure S15. DSC-curves of the second heating cycle of the covalently crosslinked metallopolymer networks **P1-Fe/Fe** (red), **P1-Co/Co** (green), **P1-Fe/Zn** (blue), and **P1-Co/Zn** (yellow) (heating rate: 10 K min^{-1}).

Thermogravimetric analysis (TGA)

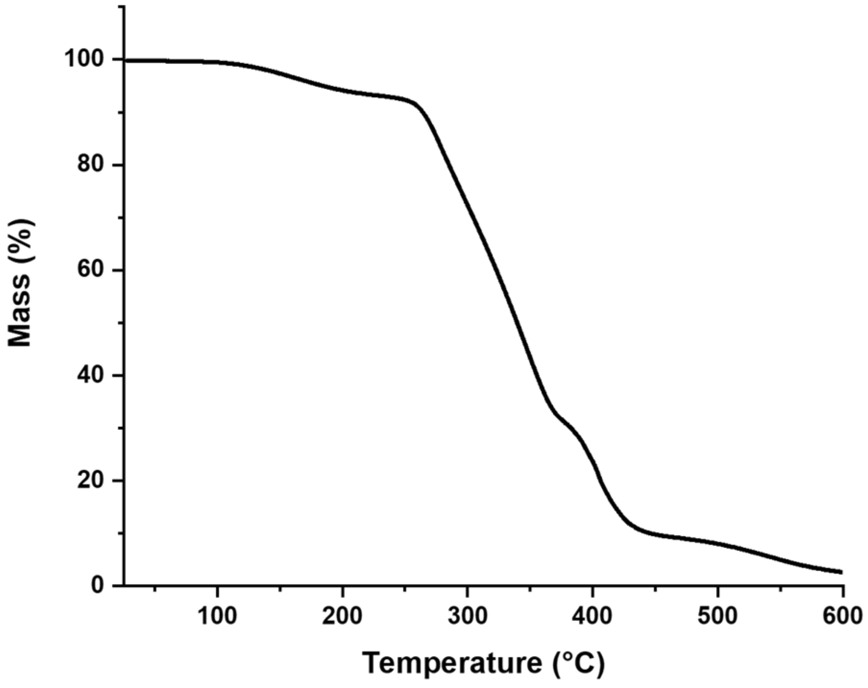

Figure S16. TGA-curve of polymer **P1** (heating rate: 10 K min^{-1}).

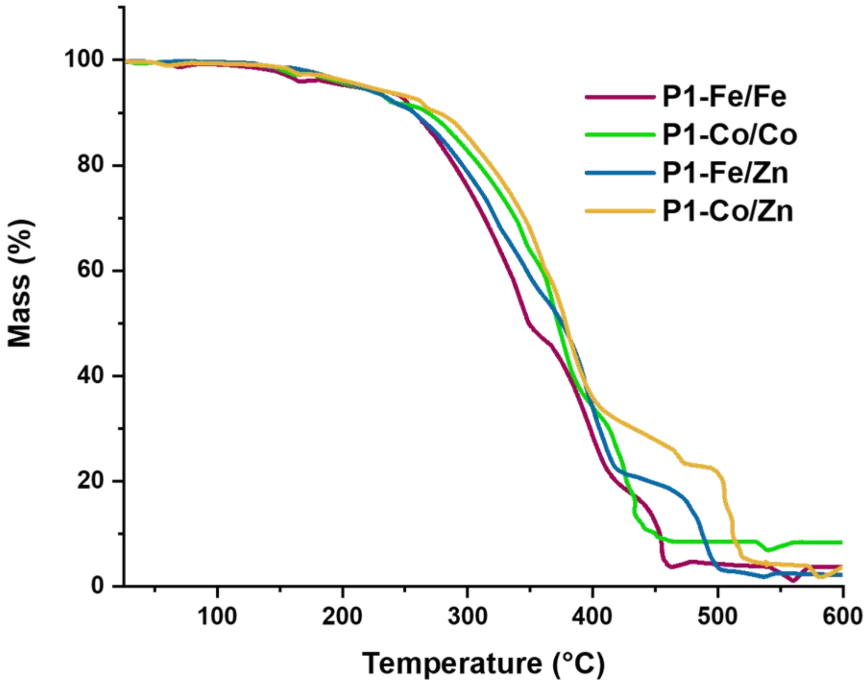

Figure S17. TGA-curves the covalently crosslinked metallopolymer networks **P1-Fe/Fe** (red), **P1-Co/Co** (green), **P1-Fe/Zn** (blue), and **P1-Co/Zn** (yellow) (heating rate: 10 K min^{-1}).

Thermo-mechanical analyses (TMA)

All TMA measurements were performed analogously to literature procedure [7].

Investigation of the dual shape-memory properties

According to literature [7], the sample was fixed into the SRF system of the rheometer. Subsequently, the temperature was increased to 150 °C (respectively 110 °C or 100 °C). Afterwards, until a total shear strain of 20% was achieved, a torsional shear strain was applied to the sample at a rate of 20% min^{-1}. In the following cooling step to 30 °C (cooling rate: 10 K min^{-1} to 2 K min^{-1}), the torsional shear strain of 20% was kept constant. Afterwards, the stress was released from the sample while keeping 30 °C. Finally, the sample was heated again to the initial temperature of 150 °C, respectively 110 °C or 100 °C, (heating rate: 10 K min^{-1}) followed by an annealing step at this temperature. The results are summarized in **Table S4**.

Table S4. Results of the thermo mechanical analysis for the investigation of the dual shape-memory properties of the metallopolymer networks **P1-Fe/Fe**, **P1-Co/Co**, **F1-Fe/Zn,** and **P1-Co/Zn** to **P3** and **P1-I** to **P3-I** at a switching temperature of 150 °C or 110 °C.

**Sample**	T_{sw} (°C)	γ_A (%)	$\gamma_{B,\,load}$ (%)	γ_B (%)	$\gamma_{A,\,rec}$ (%)
**P1-Fe/Fe**	150	0	20	20	2.0
	110	0	20	20	3.1
	100	0	20	20	2.1
**P1-Co/Co**	150	0	20	20	2.6
	110	0	20	20	2.7
	100	0	20	20	6.0
**P1-Fe/Zn**	150	0	20	20	2.5
	110	0	20	20	4.8
**P1-Co/Zn**	150	0	20	20	0.7
	110	0	20	20	3.9

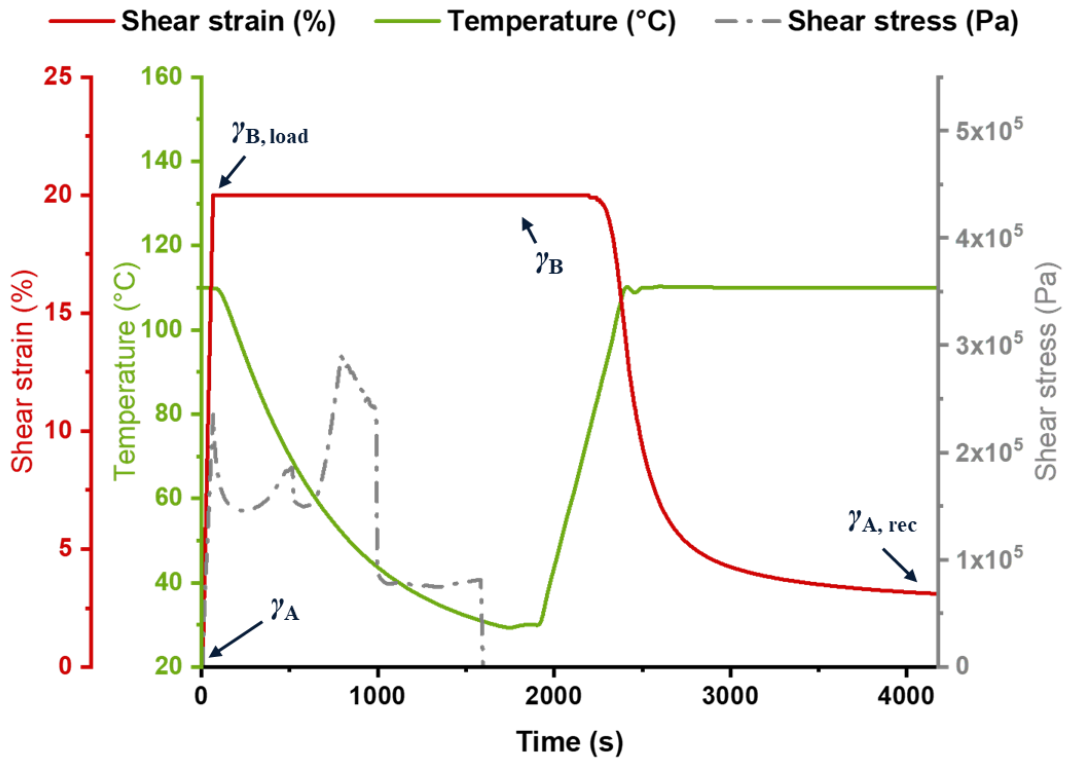

Figure S18. TMA plot of the sample **P1-Fe/Fe** with a switching temperature of 110 °C.

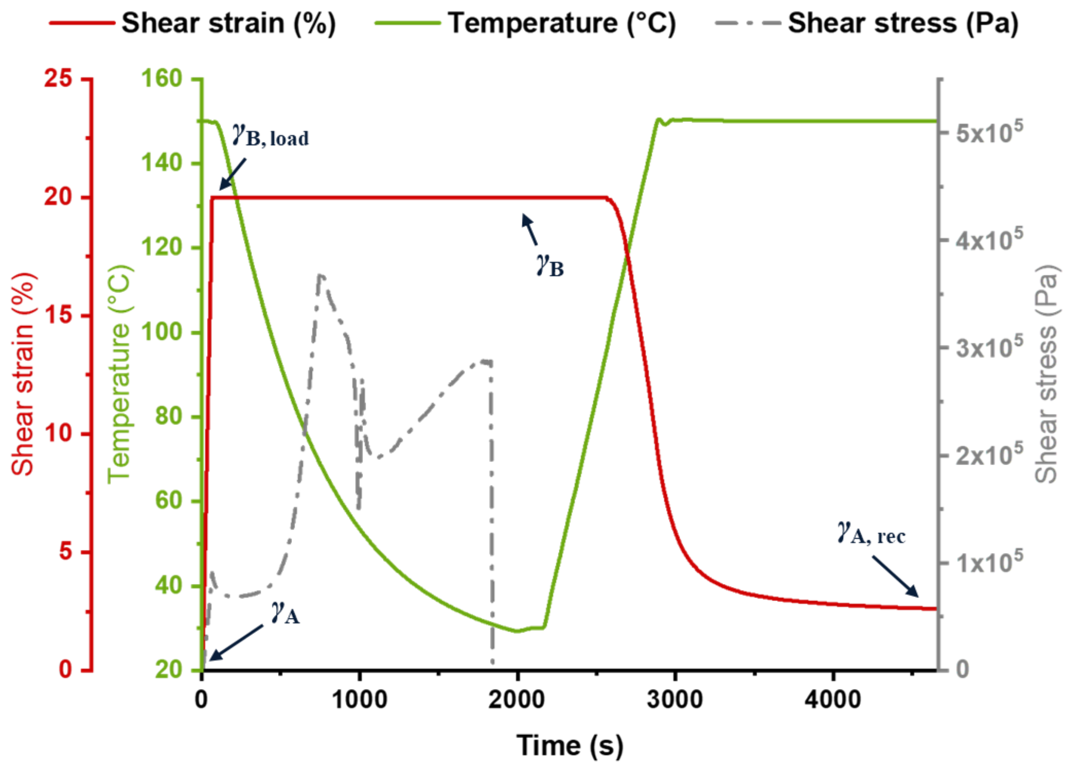

Figure S19. TMA plot of the sample **P1-Co/Co** with a switching temperature of 150 °C.

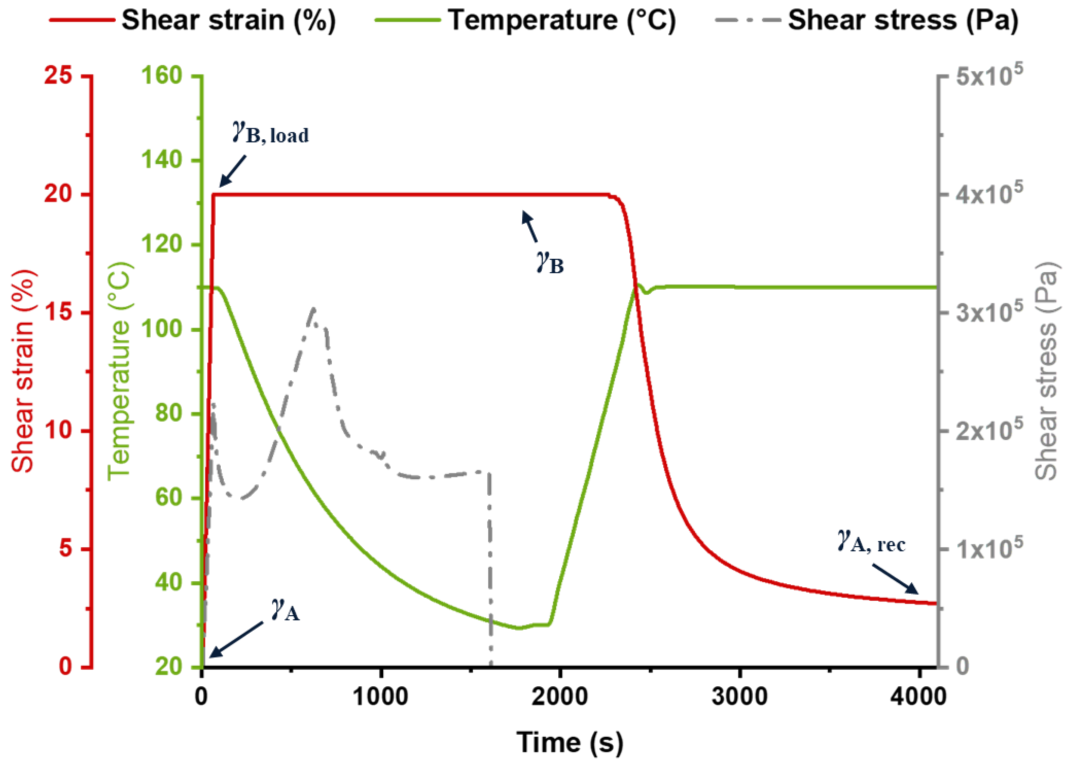

Figure S20. TMA plot of the sample **P1-Co/Co** with a switching temperature of 110 °C.

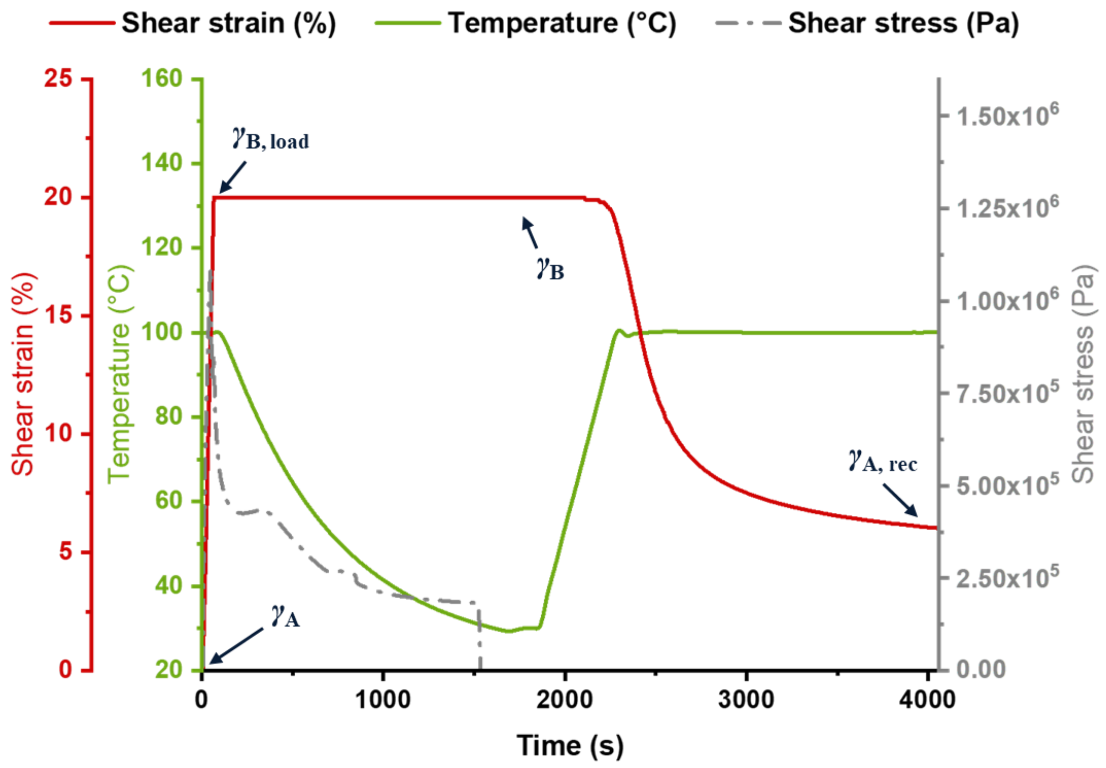

Figure S21. TMA plot of the sample **P1-Co/Co** with a switching temperature of 100 °C.

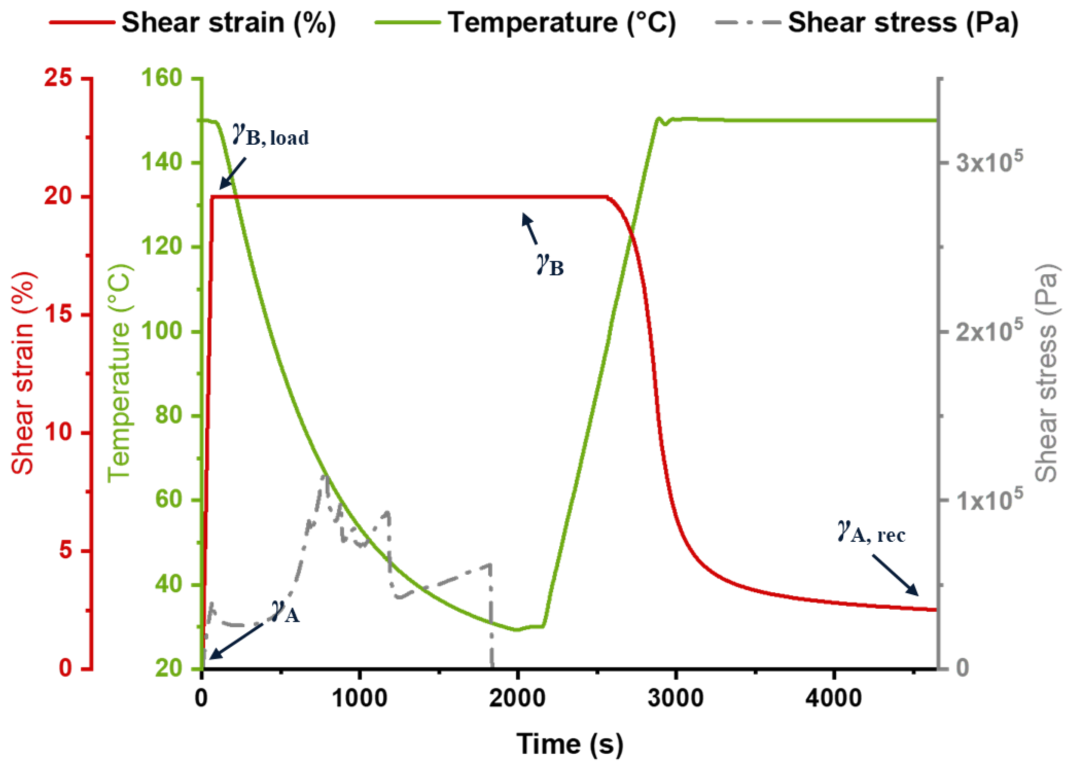

Figure S22. TMA plot of the sample **P1-Fe/Zn** with a switching temperature of 150 °C.

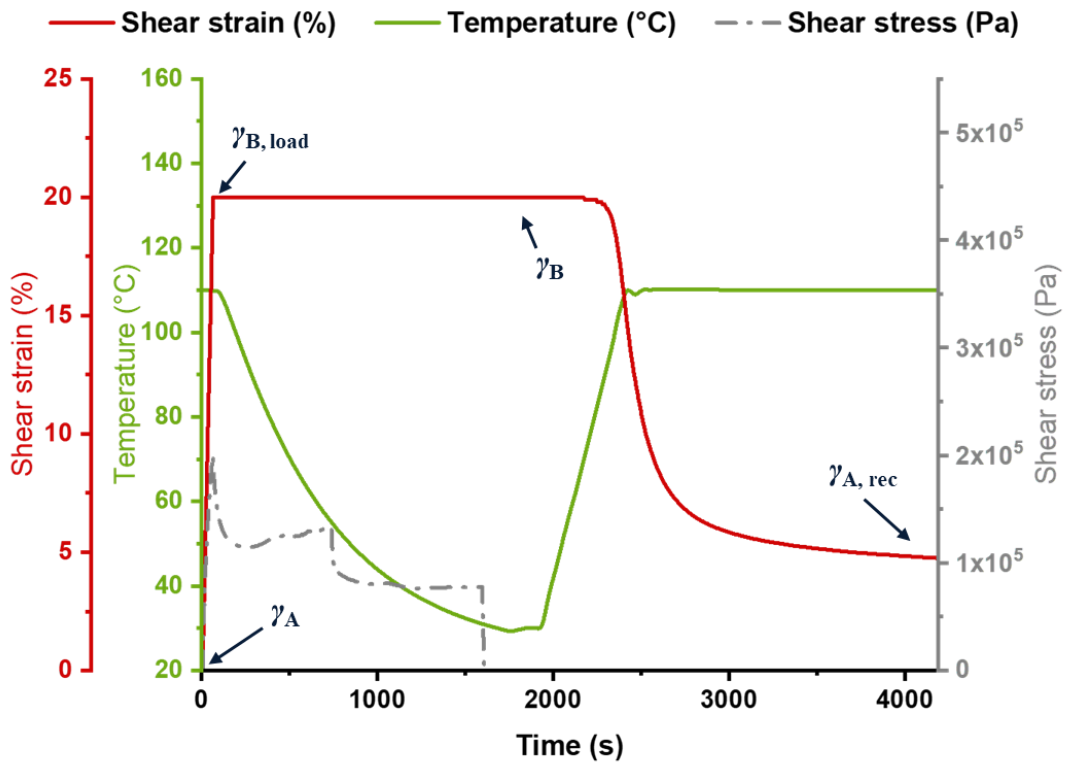

Figure S23. TMA plot of the sample **P1-Fe/Zn** with a switching temperature of 110 °C.

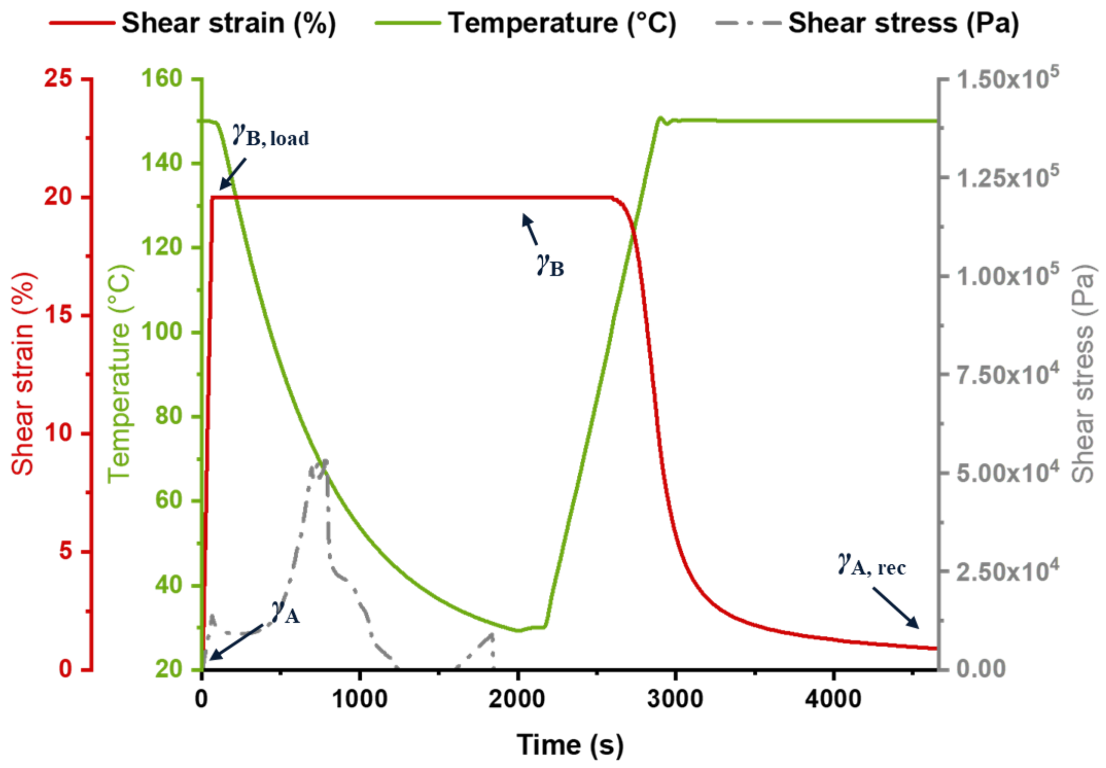

Figure S24. TMA plot of the sample **P1-Co/Zn** with a switching temperature of 150 °C.

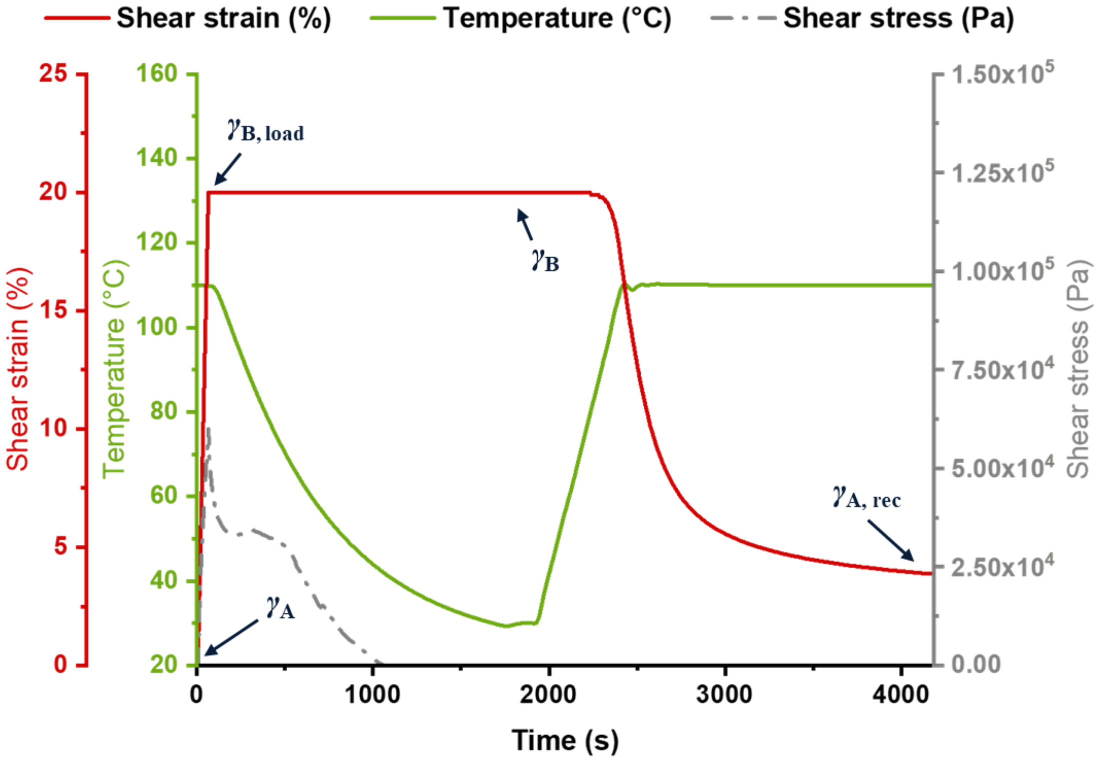

Figure S25. TMA plot of the sample **P1-Co/Zn** with a switching temperature of 110 °C.

Investigation of the triple shape-memory properties

According to a literature procedure [7], the sample was firstly fixed into the SRF system of the rheometer. Subsequently, a torsional shear strain was applied to the sample at a rate of 20% min^{-1} until a total deformation of 20% was achieved. In the following cooling step to 110 °C, respectively 100 °C (cooling rate: 10 to 2 K min^{-1}), the shear strain of 20% was kept constant. Afterwards, at 110 °C, respectively 100 °C, the stress was released from the sample, followed by an annealing step at this temperature. Subsequently, a torsional shear strain was applied to the sample at a rate of 20% min^{-1} until a total deformation of 40% was reached. The sample was cooled to 25 °C while holding the shear strain constant at 40%. After the temperature reached 30 °C, the torsional shear stress was fully removed from the sample. The next step was the annealing and, thus, the sample was heated again to 110 °C, respectively 100 °C (heating rate: 10 K min^{-1}). The temperature was kept constant for 15 minutes. Finally, the sample was again heated to the initial temperature of 150 °C (heating rate: 10 K min^{-1}) followed by an annealing step. The results are summarized in **Table S5**.

Table S5. Results of the thermo mechanical analysis for the investigation of the triple shape-memory properties of the metallopolymer networks **P1-Fe/Fe**, **P1-Co/Co**, **F1-Fe/Zn,** and **P1-Co/Zn** to **P3** and **P1-I** to **P3-I** (T_{sw1} = 150 °C and T_{sw2} = 110 °C.

Sample	T_{sw} (°C)	γ_A (%)	$\gamma_{B,\,load}$ (%)	γ_B (%)	$\gamma_{C,\,load}$ (%)	γ_C (%)	$\gamma_{B,\,rec}$ (%)	$\gamma_{A,\,rec}$ (%)
**P1-Fe/Fe**	150 and 110<br>150 and 100	0<br>0	20<br>20	15.3<br>17.4	40<br>40	40<br>40	16.1<br>20.0	1.7<br>2.8
**P1-Co/Co**	150 and 110<br>150 and 100	0<br>0	20<br>20	13.9<br>16.6	40<br>40	40<br>40	15.6<br>18.4	3.5<br>1.1
**P1-Fe/Zn**	150 and 110	0	20	17.3	40	40	20.5	6.0
**P1-Co/Zn**	150 and 110	0	20	18.1	40	40	20.5	0.7

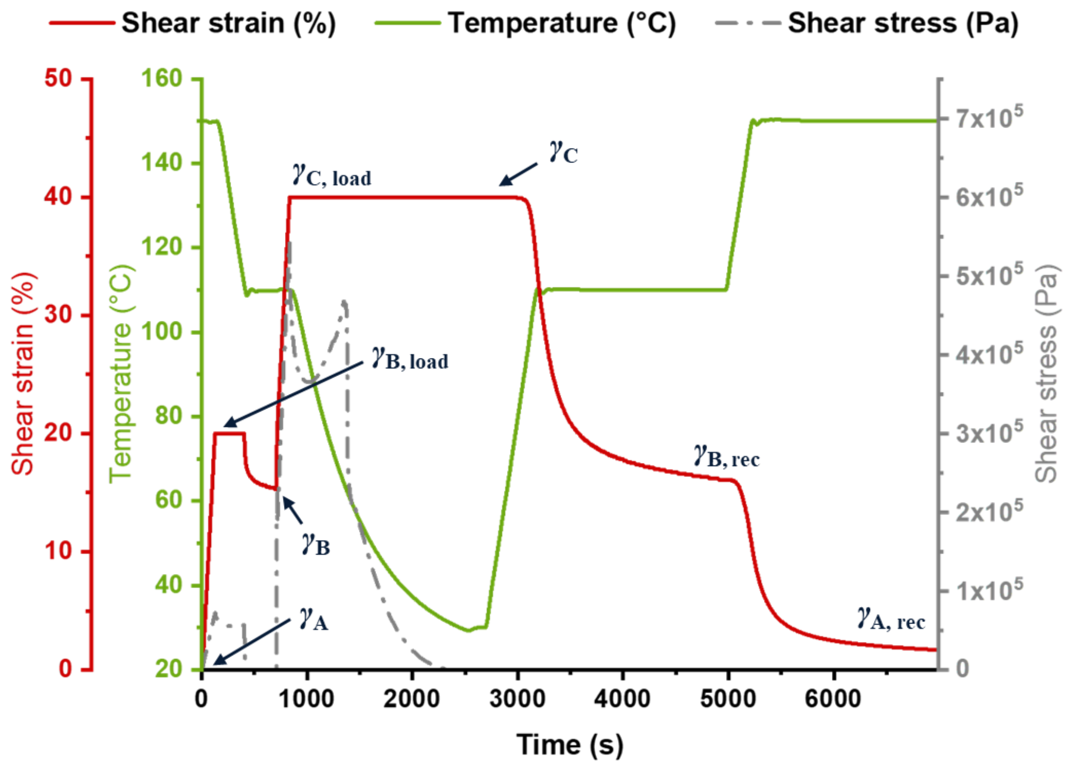

Figure S26. TMA plot of the sample **P1-Fe/Fe** for the investigation of the triple shape-memory properties (T_{sw1} = 150 °C and T_{sw2} = 110 °C).

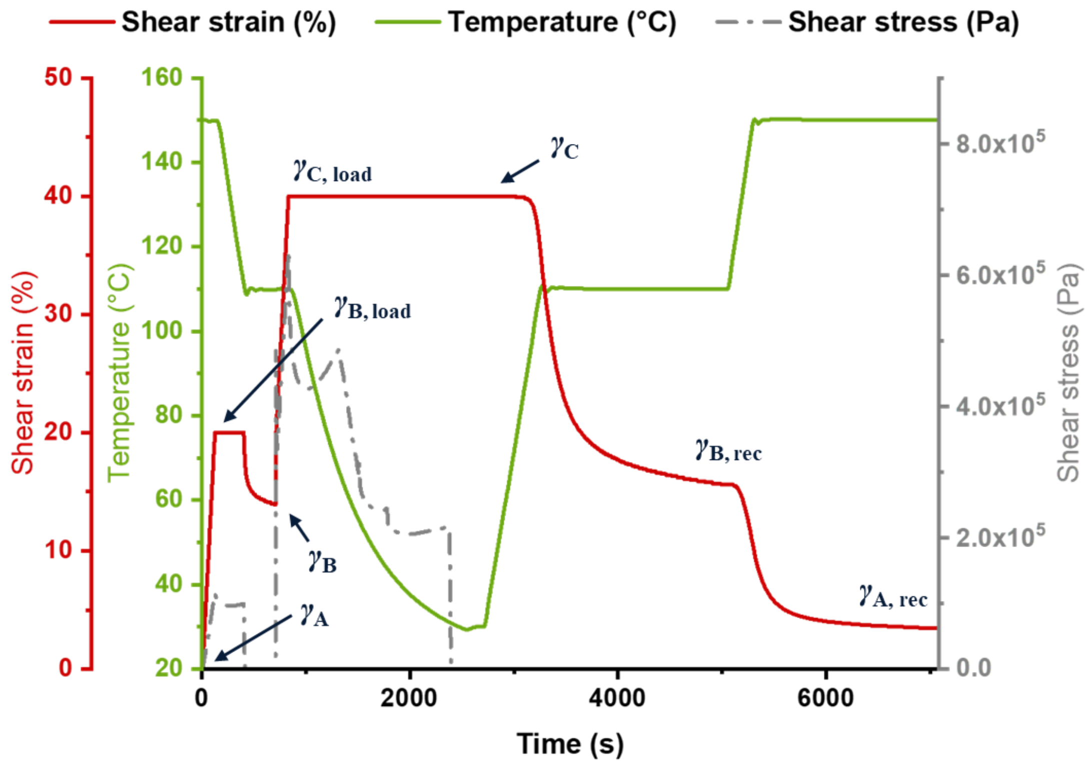

Figure S27. TMA plot of the sample **P1-Co/Co** for the investigation of the triple shape-memory properties (T_{sw1} = 150 °C and T_{sw2} = 110 °C).

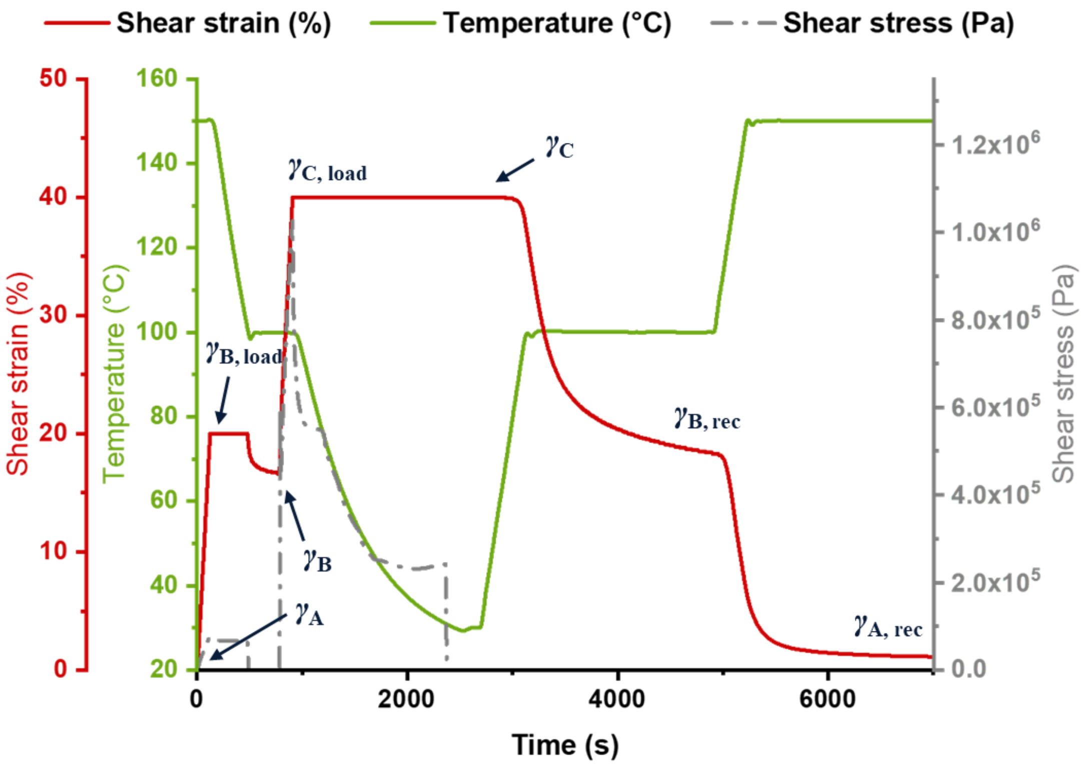

Figure S28. TMA plot of the sample **P1-Co/Co** for the investigation of the triple shape-memory properties (T_{sw1} = 150 °C and T_{sw2} = 100 °C).

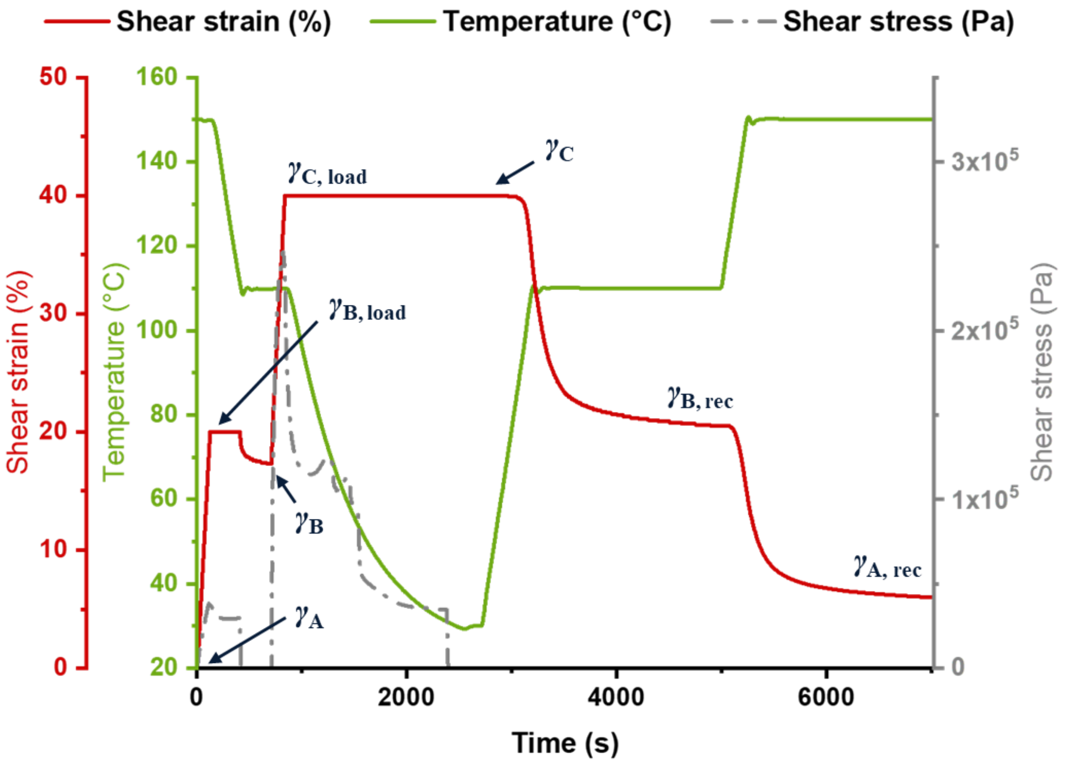

Figure S29. TMA plot of the sample **P1-Fe/Zn** for the investigation of the triple shape-memory properties (T_{sw1} = 150 °C and T_{sw2} = 110 °C).

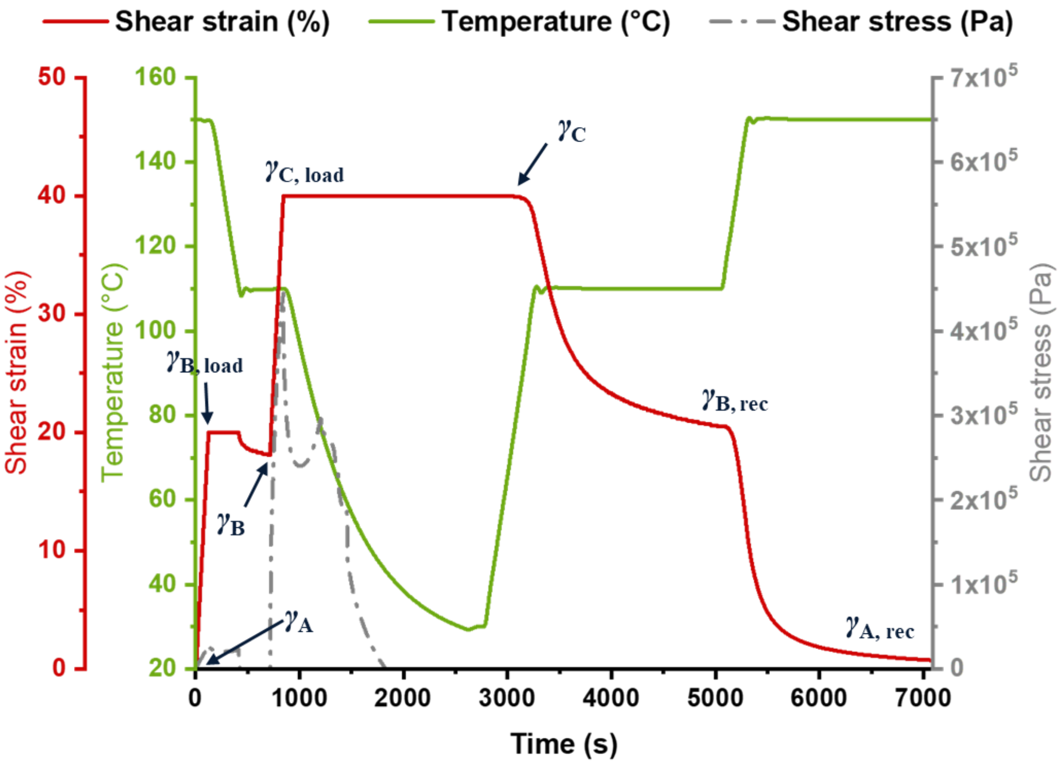

Figure S30. TMA plot of the sample **P1-Co/Zn** for the investigation of the triple shape-memory properties (T_{sw1} = 150 °C and T_{sw2} = 110 °C).

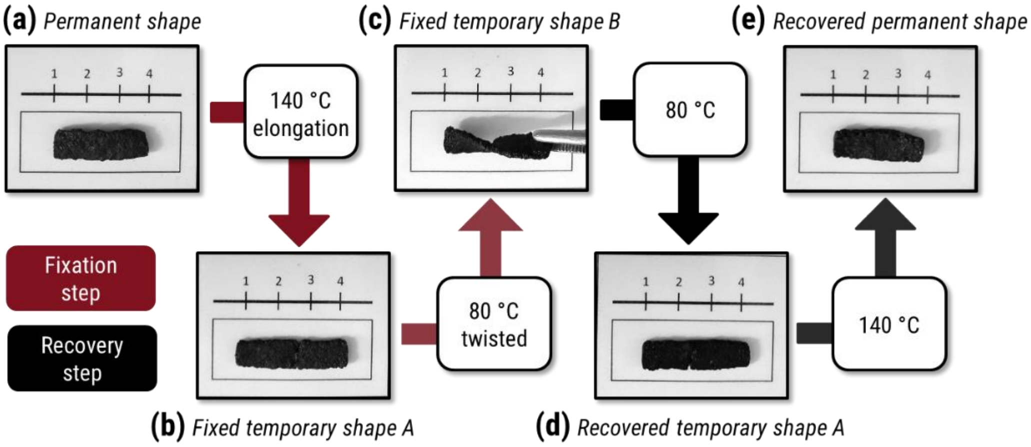

Figure S31. Photo series of the triple shape-memory test of the covalently crosslinked metallopolymer network **P1-Fe/Fe**.

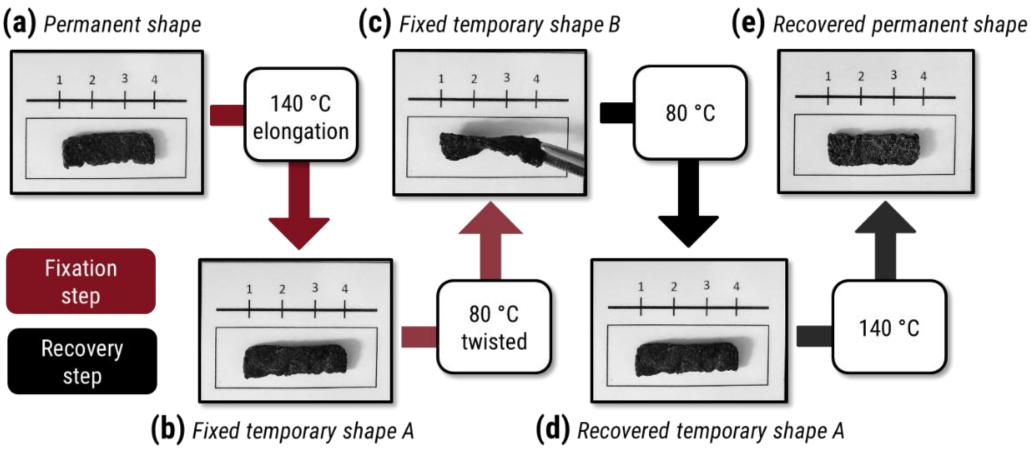

Figure S32. Photo series of the triple shape-memory test of the covalently crosslinked metallopolymer network **P1-Co/Co**.

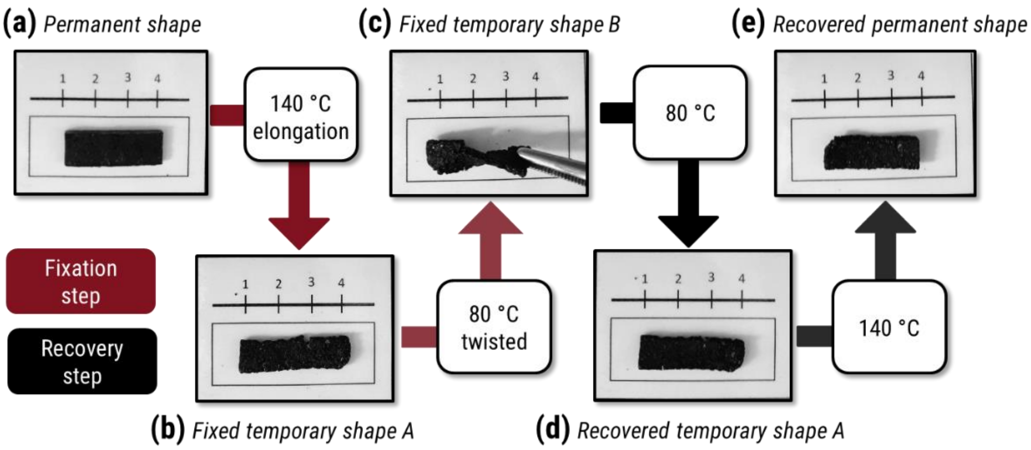

Figure S33. Photo series of the triple shape-memory test of the covalently crosslinked metallopolymer network **P1-Fe/Zn**.

FT-Raman spectroscopy

FT-Raman spectroscopy measurements were performed on a Multispec Fourier-transform Raman-Spectrometer (Bruker Corporation, Billerica, Massachusetts, United States of America) in the range between 100 and 4000 cm^{-1} with a spectral resolution of 4 cm^{-1}. The Raman excitation light at 1064 nm was provided by a Nd:YAG laser (Klastech Den-iCAFC-LC-3/40, Dortmund, Germany). The laser power at the sample was set to 100 mW and 512 accumulated scans were recorded for each sample to improve the signal-to-noise ratio.

The raw spectra were processed for further evaluation using R 4.0.2 [8]: The spectra were restricted to the wavenumber of interest (e.g., 400 to 1800 cm^{-1}), background corrected using the SNIP-algorithm (iterations: 60, order: 3, smoothing window: 3) [9], and normalized using Euclidean vector norm.

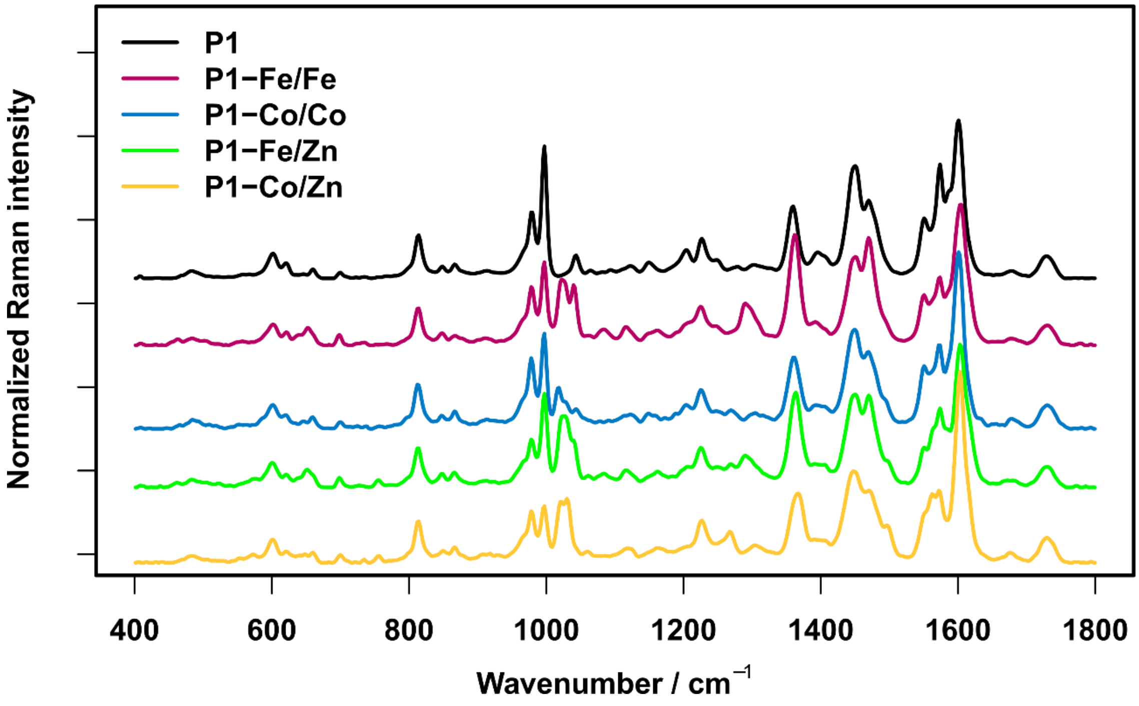

Figure S34. Raman Spectra of **P1** and the metallopolymers **P1-Fe/Fe**, **P1-Co/Co, P1-Fe/Zn,** and **P1-Co/Zn** in the wavenumber region between 400 and 1800 cm^{-1}.

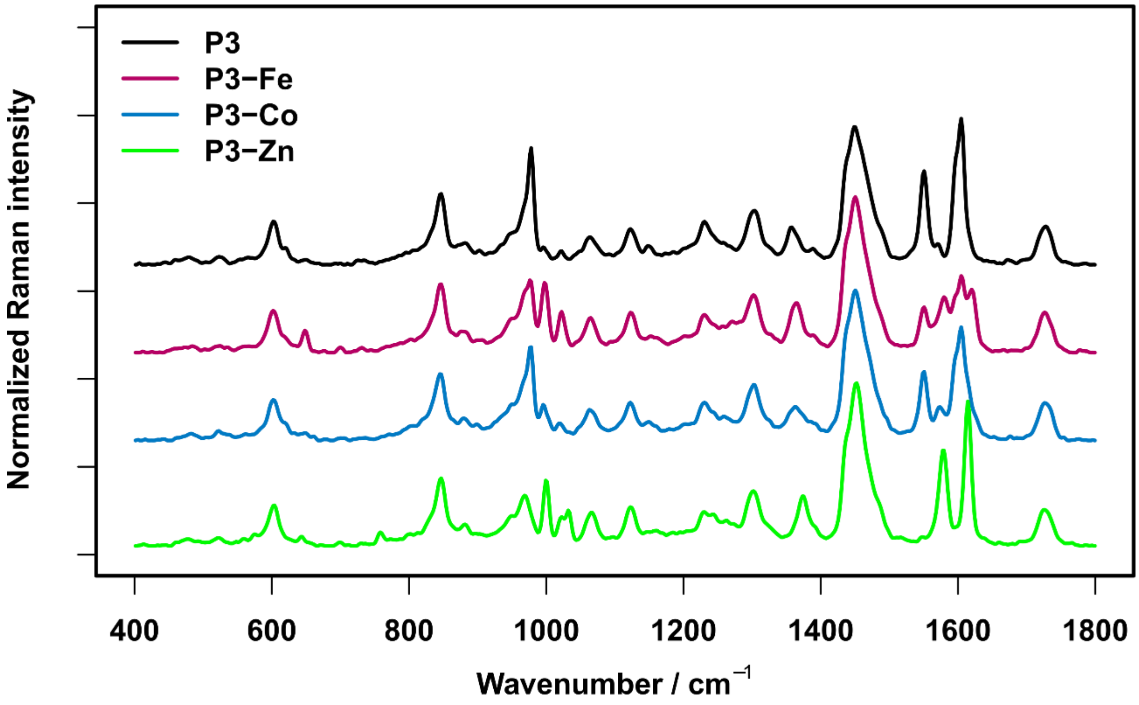

Figure S35. Raman Spectra of **P3** and the metallopolymers **P3-Fe**, **P3-Co,** and **P3-Co** in the wavenumber region between 400 and 1800 cm^{-1}.

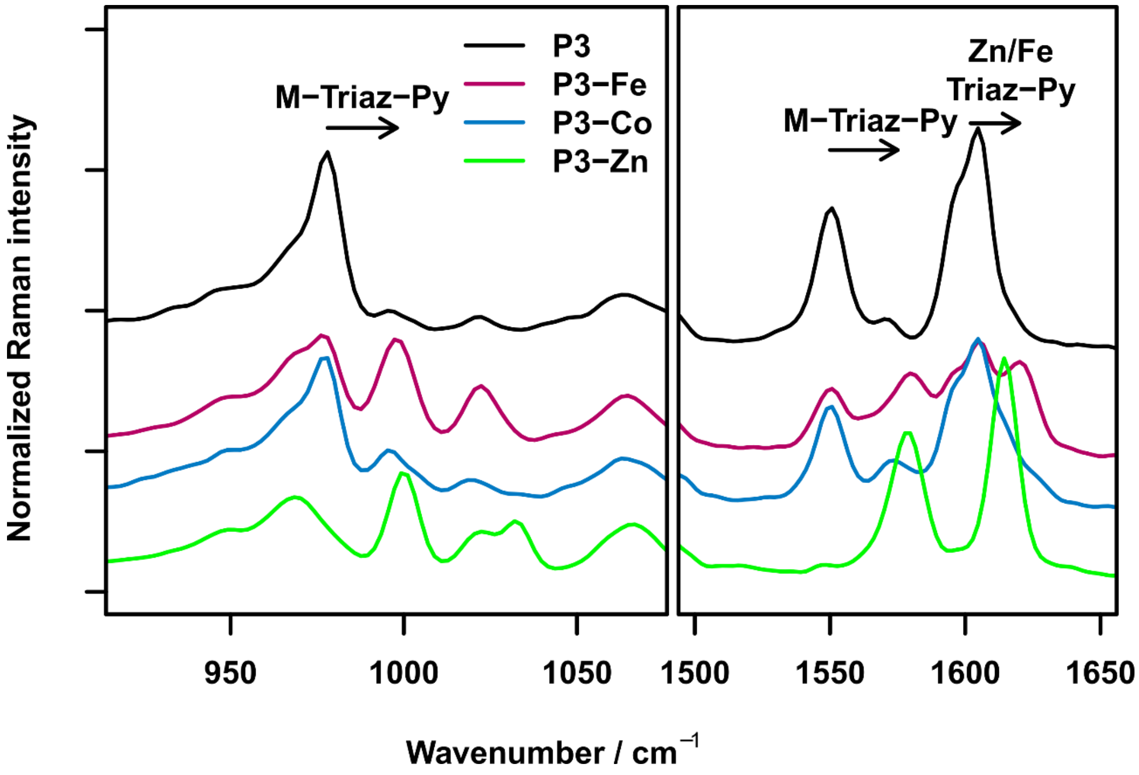

Figure S36. Raman Spectra of **P3** and the metallopolymers **P3-Fe**, **P3-Co,** and **P3-Zn** in the regions of interest (920 to 1060 cm^{-1} and 1500 to 1650 cm^{-1}) for complex formation. The arrows indicate band shifts of marker bands of **Triaz-Py** upon metal binding.

References

1. Bode, S.; Zedler, L.; Schacher, F.H.; Dietzek, B.; Schmitt, M.; Popp, J.; Hager, M.D.; Schubert, U.S. Self-healing polymer coatings based on crosslinked metallosupramolecular copolymers. *Adv. Mater.* **2013**, *25*, 1634–1638.
2. Happ, B.; Friebe, C.; Winter, A.; Hager, M.D.; Hoogenboom, R.; Schubert, U.S. 2-(1 h-1,2,3-Triazol-4-yl)-pyridine ligands as alternatives to 2,2′-bipyridines in ruthenium(ii) complexes. *Chem. Asian J.* **2009**, *4*, 154–163.
3. Happ, B.; Pavlov, G.M.; Perevyazko, I.; Hager, M.D.; Winter, A.; Schubert, U.S. Induced charge effect by co(ii) complexation on the conformation of a copolymer containing a bidentate 2-(1,2,3-triazol-4-yl)pyridine chelating unit. *Macromol. Chem. Phys.* **2012**, *213*, 1339–1348.
4. Meurer, J.; Hniopek, J.; Zechel, S.; Enke, M.; Vitz, J.; Schmitt, M.; Popp, J.; Hager, M.D.; Schubert, U.S. Shape-memory metallopolymer networks based on a triazole-pyridine ligand. *Polymers* **2019**, *11*.
5. Meurer, J.; Bätz, T.; Hniopek, J.; Zechel, S.; Schmitt, M.; Popp, J.; Hager, M.D.; Schubert, U.S. Dual crosslinked metallopolymers using orthogonal metal complexes as rewritable shape-memory polymers. *J. Mater. Chem. A* **2021**, *9*, 15051–15058.
6. Meurer, J.; Hniopek, J.; Bätz, T.; Zechel, S.; Enke, M.; Vitz, J.; Schmitt, M.; Popp, J.; Hager, M.D.; Schubert, U.S. Shape-memory metallopolymers based on two orthogonal metal–ligand interactions. *Adv. Mater.* **2021**, *33*, 2006655.
7. Meurer, J.; Rodriguez Agudo, J.A.; Zechel, S.; Hager, M.D.; Schubert, U.S. Quantification of triple-shape memory behavior of polymers utilizing tension and torsion. *Macromol. Chem. Phys.* **2021**, *222*, 2000462.
8. Team, R.C. R: A language and environment for statistical computing. R foundation for statistical computing, vienna, austria. https://www.R-project.Org/ **2021**.
9. Ryan, C.G.; Clayton, E.; Griffin, W.L.; Sie, S.H.; Cousens, D.R. Snip, A statistics-sensitive background treatment for the quantitative analysis of pixe spectra in geoscience applications. *Nuclear Instruments and Methods in Physics Research Section B: Beam Interactions with Materials and Atoms* **1988**, *34*, 396–402.
